# Unique Biofilm Signature, Drug Susceptibility and Decreased Virulence in *Drosophila* through the *Pseudomonas aeruginosa* Two-Component System PprAB

**DOI:** 10.1371/journal.ppat.1003052

**Published:** 2012-11-29

**Authors:** Sophie de Bentzmann, Caroline Giraud, Christophe S. Bernard, Virginie Calderon, Friederike Ewald, Patrick Plésiat, Cathy Nguyen, Didier Grunwald, Ina Attree, Katy Jeannot, Marie-Odile Fauvarque, Christophe Bordi

**Affiliations:** 1 Laboratoire d'Ingénierie des Systèmes Macromoléculaires, CNRS - Aix Marseille University, Marseille, France; 2 Université de Toulouse, UPS, Laboratoire de Microbiologie et Génétique Moléculaire, Toulouse, France, and CNRS, LMGM, Toulouse, France; 3 Laboratoire de Bactériologie, Faculté de Médecine-Pharmacie, Université de Franche-Comté, Besançon, France; 4 TAGC U928 case 928, Marseille, France; 5 CNRS ERL5261 “Bacterial Pathogenesis and Cellular Responses,” CEA, iRTSV, Biologie du Cancer et de l'Infection, Grenoble, France and INSERM, UMR-S 1036, Grenoble, France and UJF-Grenoble I, UMR-S 1036, Grenoble, France; 6 CEA, iRTSV, Biologie à Grande Echelle, Grenoble, France and INSERM, U1038, Grenoble, France and UJF-Grenoble I, U1038, Grenoble, France; Stanford University, United States of America

## Abstract

Bacterial biofilm is considered as a particular lifestyle helping cells to survive hostile environments triggered by a variety of signals sensed and integrated through adequate regulatory pathways. *Pseudomonas aeruginosa*, a Gram-negative bacterium causing severe infections in humans, forms biofilms and is a fantastic example for fine-tuning of the transition between planktonic and community lifestyles through two-component systems (TCS). Here we decipher the regulon of the *P. aeruginosa* response regulator PprB of the TCS PprAB. We identified genes under the control of this TCS and once this pathway is activated, analyzed and dissected at the molecular level the PprB-dependent phenotypes in various models. The TCS PprAB triggers a hyper-biofilm phenotype with a unique adhesive signature made of BapA adhesin, a Type 1 secretion system (T1SS) substrate, CupE CU fimbriae, Flp Type IVb pili and eDNA without EPS involvement. This unique signature is associated with drug hyper-susceptibility, decreased virulence in acutely infected flies and cytotoxicity toward various cell types linked to decreased Type III secretion (T3SS). Moreover, once the PprB pathway is activated, decreased virulence in orally infected flies associated with enhanced biofilm formation and dissemination defect from the intestinal lumen toward the hemolymph compartment is reported. PprB may thus represent a key bacterial adaptation checkpoint of multicellular and aggregative behavior triggering the production of a unique matrix associated with peculiar antibiotic susceptibility and attenuated virulence, a particular interesting breach for therapeutic intervention to consider in view of possible eradication of *P. aeruginosa* biofilm-associated infections.

## Author Summary

We unraveled that once the two-component system PprAB regulatory pathway is activated, *Pseudomonas aeruginosa* displays a unique hyper-biofilm phenotype due to a molecular signature combining a T1SS high molecular weight substrate, BapA, fimbriae of the chaperone-usher pathway, Type IVb pili and eDNA. Originally, this particular hyper-biofilm that is not strictly dependent on Psl or Pel exopolysaccharide (EPS) synthesis displays increased drug susceptibility, in contrary to previously reported biofilm lifestyle associated with increased resistance to antibiotics. PprB-dependent hyper-biofilm was also observed on intestinal mucosa of orally infected *Drosophila* flies in which it also displays a reduced capacity to cross the epithelial barrier from the intestinal lumen toward the hemolymph that consequently resulted in a reduced capacity to kill flies. Furthermore, constitutive activation of this PprB regulatory pathway triggers a reduced secretion of T3SS effectors which may account for the decreased virulence observed in epithelial and macrophage lineages and in acute *Drosophila* infections induced by septic injury. We appended in this study pieces of regulatory and molecular data that highlight the possibility to combat infections due to *P. aeruginosa*-biofilm with particular matrix.

## Introduction

Almost all bacterial species can live in sessile communities called biofilms. Biofilms are a particular problem for human health, not only because they may cause several infectious diseases, but because they are associated with many inert surfaces, including those of medical devices for internal or external use [1] and are very difficult to eradicate. Moreover, their associated existence with infections is probably underestimated, principally due to the need for *in vivo* diagnosis [2]. A common property of biofilms is the presence of a matrix surrounding the community, shaping and protecting it, as well as, immobilizing its members. This auto-produced matrix is mainly composed of exopolysaccharides (EPS), proteins (soluble and parietal mostly assembled into structures such as fimbriae, pili and flagella), lipids and DNA, forming a hydrated polymer, the composition of which could be greatly influenced by the species forming the community as well as by environmental conditions [3]. Biofilm formation is a dynamic process passing through a four-stage universal growth cycle consisting of initiation (attachment of planktonic cells to the substrate), maturation, maintenance, and dissolution (these three being considered as post-attachment) phases. The transition between planktonic and community lifestyles in bacteria is tightly regulated and depends on environmental signals that the bacteria integrate to unfold the most appropriate response. This requires a complex regulatory network including two-component systems (TCS), transcriptional regulators, quorum sensing and post-transcriptional regulatory mechanisms [1]. Among them, the TCSs represent a predominant signaling mechanism in bacteria and are formed of a histidine kinase (HK) and a response regulator (RR) in the simplest and termed classical form.

*Pseudomonas aeruginosa*, a Gram-negative bacterium that causes severe infections in humans, can infect non-mammalian hosts by using similar virulence mechanisms as in mammalian models [4]–[7]. *P. aeruginosa* forms biofilms and is a fantastic example for fine-tuning of the transition between planktonic and community lifestyles through TCSs. The *P. aeruginosa* PAO1 genome sequence possesses 63 HK and 64 RR [8], with additional copies identified in newly sequenced genomes [9], [10]. Among them, a substantial number has been demonstrated to be involved in community lifestyle or in the transition between planktonic and community lifestyles. The molecular switch executed by the three sensors RetS/GacS/LadS is a good example of such a regulatory mechanism involving small RNAs RsmY and RsmZ. Although the GacSA TCS and the hybrid HK LadS [11] are activating biofilm formation and negatively regulating Type III secretion system (T3SS)-dependent cytotoxicity through sRNA expression, GacS is antagonized by the hybrid HK RetS [12]. Whereas RetS/LadS/GacS pathway controls RsmY and RsmZ sRNAs, the HptB signaling pathway acts solely on RsmY production and positively regulates biofilm and negatively T3SS-dependent cytotoxicity [13]. Further post-attachment reduction of *rsmZ* RNA level in biofilms, is dependent on another TCS BfiSR, which directly regulates expression of the *cafA* gene encoding RNase G acting on RsmZ stability [14]. BfiSR belongs to the three novel *P. aeruginosa* TCSs essential in regulating the transition to irreversible attachment (BfiSR, stage 1–2), maturation-1 (BfmSR, stage 2–3), and maturation-2 (MifSR, stage 3–4) during biofilm development [15]. Beside those TCSs implicating sRNA, other TCSs like the Roc1 [16] and Roc2 [17] and the four player PvrRS/RcsBC TCSs [18], probably act on the control of the intracellular levels of c-di-GMP and on fimbrial gene expression of the chaperone-usher (CU) systems CupB, CupC and CupD [19], respectively. The Pseudomonas (membrane) Permeability Regulator (PprB) gene encodes the RR of the classical TCS PprAB, with its cognate HK PprA. The PprB RR is a member of the NarL/LuxR family, with a receiver domain and a DNA-binding, helix-turn-helix (HTH) domain. It was first identified as involved in the increased permeability of the *P. aeruginosa* membrane to various antibiotics in the PAK genetic background [20]. It was interestingly characterized as controlling directly and positively the expression of two molecular systems helping this bacterium to form biofilm [21], which are Type IVb Flp pili [22] and CU CupE fimbriae [23].

The present study was dedicated to the determination of the PprB regulon using transcriptomic analysis. From the common target genes differentially regulated in the strain overproducing PprB versus the *pprB* mutant, we identified a limited number of genes that we then validated using transcriptional fusions, RT-qPCR and electrophoretic mobility shift assay (EMSA) approaches. Among them, we identified genes encoding a Type 1 secretion system (T1SS) secreting an adhesin BapA, and the *Pseudomonas* quinolone signal (PQS) system. The molecular analysis of the global behavior associated with the activation of the PprB pathway was further conducted. Beside *in vitro* flow cell and cellular models, we tested the contribution of the PprB pathway to *in vivo* biofilm formation and bacterial virulence in *Drosophila melanogaster* flies. In this model, septic injury provokes death by septicemia in a day, mimicking an acute infection whereas virulent bacteria present in contaminated food induce gut chronic infection and death in several days by crossing the gut epithelial barrier and invading the hemocoele [24]–[27].

Under PprB-activated pathway conditions, hyper-biofilm phenotype relied on a unique combination of Type IVb pili, CupE fimbriae, BapA adhesin and eDNA rather than on Pel and Psl, the two major EPS of non-mucoid strains. Activation of the PprB regulatory pathway is associated with drug hyper susceptibility particularly in biofilms and attenuated virulence in both acute and chronic *Drosophila* infection models. Thus, the response regulator PprB is a new player of the key regulators of *P. aeruginosa* participating to the transition between planktonic and community lifestyles.

## Results

Activation of a TCS regulatory pathway can be frequently assumed by overexpression of the RR gene onto a replicative plasmid. Determination of the PprB regulon was thus performed using comparison of transcriptomic profiles of the PAO1*attB*::*cupE-lacZ* strain carrying the empty vector pMMB67-HE (reference condition) and of the PAO1*attB*::*cupE-lacZ* strain carrying the pMMB*pprB* vector (inducing condition) (Experiment 1), and of the PAO1Δ*pprBattB*::*cupE-lacZ* strain (reference condition) and of the PAO1*attB*::*cupE-lacZ* strain (inducing condition) (Experiment 2). To override the requirement for antibiotic use to maintain plasmids in phenotypic analysis, all experiments aiming at the characterization of the PprB-dependent phenotypes were further conducted in the PprBK (PAO1_LB_Tn10) [23] genetic background. This strain was obtained in a previous wide-genome transposon mutagenesis conducted to obtain *cupE* regulators. It possesses the Mariner transposon located upstream from the *fppA* gene, with the internal p*tac* promoter driving the expression of downstream *pprB* gene, as checked by RT-qPCR. We checked that in this strain, target genes overlap the ones presented in Experiment 1 (data not shown).

### Analysis of transcriptional profiles

In order to identify new genes potentially regulated by the TCS PprAB, we designed MGPA arrays (Agilent) (see supplemental experimental procedures) and carried out microarray experiments. Specific genome-wide expression profiles were obtained in two sets of experiments as described above. Applying selective criteria (see Supplemental experimental procedures), 130 and 56 genes (see supplementary Tables S1 and S2) were found to be differentially expressed in Experiments 1 and 2, respectively. To focus on genes belonging to the PprB regulon, genes conversely expressed in common in these two sets of experiments were selected after intensive analytical treatment and a final list of 35 genes has been established (Table 1). Among them, 18 belong to the *flprcptad* (13) and the *cupE* (5) loci already described to be under the direct control of the TCS PprAB [22], [23], a result which validated our approach (Fig. S1). The *flprcptad* locus refers to a DNA region constituted of five transcriptional units that encodes the Flp Type IVb pilus assembly machinery and contains the *pprA* and *pprB* genes, the two *tcs* genes positively regulating all the other genes encoding the pilus machinery [22].

**Table 1:** List of genes commonly and conversely regulated in microarray experiments (1 : PAO1*attB*::*cupE-lacZ*/pMMB*pprB* strain vs PAO1*attB*::*cupE-lacZ*/pMMB67HE strain, 2 : PAO1*attB*::*cupE-lacZ* strain vs PAO1Δ*pprBattB*::*cupE-lacZ* strain).

PA number (Locus tag)	SwissProt	Function	Fold change in experiment 1^a^	Fold change in experiment 2^a^
PA0998	Q9I4X1	Homologous to beta-keto-acyl-acyl-carrier protein synthase PqsC	+3.45	−3.27
PA0999	P20582	3-oxoacyl-[acyl-carrier-protein] synthase III PqsD	+4.02	−3.6
PA1000	P20581	Quinolone signal response protein PqsE	+3.66	−3.43
PA1001	P09785	Anthranilate synthase component I PhnA	+3.74	−3.45
PA1002	P09786	Anthranilate synthase component II PhnB	+3.59	−3.4
PA1213	Q9I4C5	putative clavaminic acid synthetase	+2	−2.2
PA1214	Q9I4C4	Putative sparagine synthase	+2.16	−2.59
PA1216	Q9I4C2	Hypothetical protein	+2.39	−2.15
PA1217	Q9I4C1	Probable 2-isopropylmalate synthase	+2.39	−2.53
PA1218	Q9I4C0	Hypothetical protein	+2.21	−2.6
PA1874	Q9I2M3	Large uncharacterized protein BapA	+25.72	−12.64
PA1875	Q9I2M2	Probable outer membrane protein of T1SS transporter BapB	+28.81	−9.17
PA1876	Q9I2M1	probable ATP-binding protein of T1SS transporter BapC	+19.53	−6.41
PA1877	Q9I2M0	probable fusion protein of T1SS transporter BapD	+16.94	−7.11
PA1914	Q9I2J0	HvnA	+31.86	−3.87
PA3662	Q9HXX7	Hypothetical protein	−9.5	+3.73
PA4293	Q9HWA7	Histidine kinase PprA	+11.5	−4.45
PA4294	Q9HWA6	Pseudopilin TadF	+15.68	−4.04
PA4296	Q9HWA4	Response regulator PprB	+32.23	−38.5
PA4297	Q9HWA3	TadG	+3.86	−20.24
PA4298	Q9HWA2	Hypothetical protein	+4.35	−10.76
PA4299	Q9HWA1	TadD	+5.13	−16.84
PA4300	Q9HWA0	PilC-like protein TadC	+5.51	−15.8
PA4301	Q9HW99	PilC-like protein TadB	+4.46	−11.05
PA4302	Q9HW98	ATPase TadA	+5.49	−19.17
PA4303	Q9HW97	TadZ	+4.36	−9.95
PA4304	Q9HW96	Secretin RcpA	+5.33	−13.45
PA4305	Q9HW95	RcpC	+5.33	−17
PA4306	Q9HW94	Pilin subunit Flp	+5.6	−39
PA4648	Q9HVE4	Fimbrial subunit CupE1	+5.67	−7.03
PA4649	Q9HVE3	Fimbrial subunit CupE2	+7.98	−11.85
PA4650	Q9HVE2	Fimbrial subunit CupE3	+6.75	−7.45
PA4651	Q9HVE1	Chaperone CupE4	+7.74	−11.7
PA4652	Q9HVE0	Usher CupE5	+2.12	−3.53
PA5287	Q9HTR7	Ammonium transporter AmtB	+2.28	−2.21

a fold changes correspond to mean values of the four replicates performed for each condition.

Seventeen new candidates belonging to 6 different loci were identified (Fig. S1) including i/ the *pqsA-phnA* locus involved in the production of PQS, ii/ the *PA1221-1210* locus encoding proteins of unknown functions, iii/ the *PA1874-1877* locus which encodes a large uncharacterized protein and a T1SS transporter described as involved in antibiotic resistance [28], that we renamed *bapABCD* locus, iv/ the *PA1914* gene encoding a putative homologue of HvnA protein of *Vibrio fischeri* [29], that we renamed *hvnA*, v/ the *PA3662* gene encoding a protein with unknown function and vi/ the *PA5287* gene encoding the ammonium transporter AmtB, forming an operon with the gene *glnK* encoding the nitrogen regulatory protein P-II 2. All these new identified genes, except *PA3662*, were positively regulated by the RR PprB (upregulated under PprB overproduction conditions and downregulated in the *pprB* mutant).

### Validation of target genes

**Figure 1:**
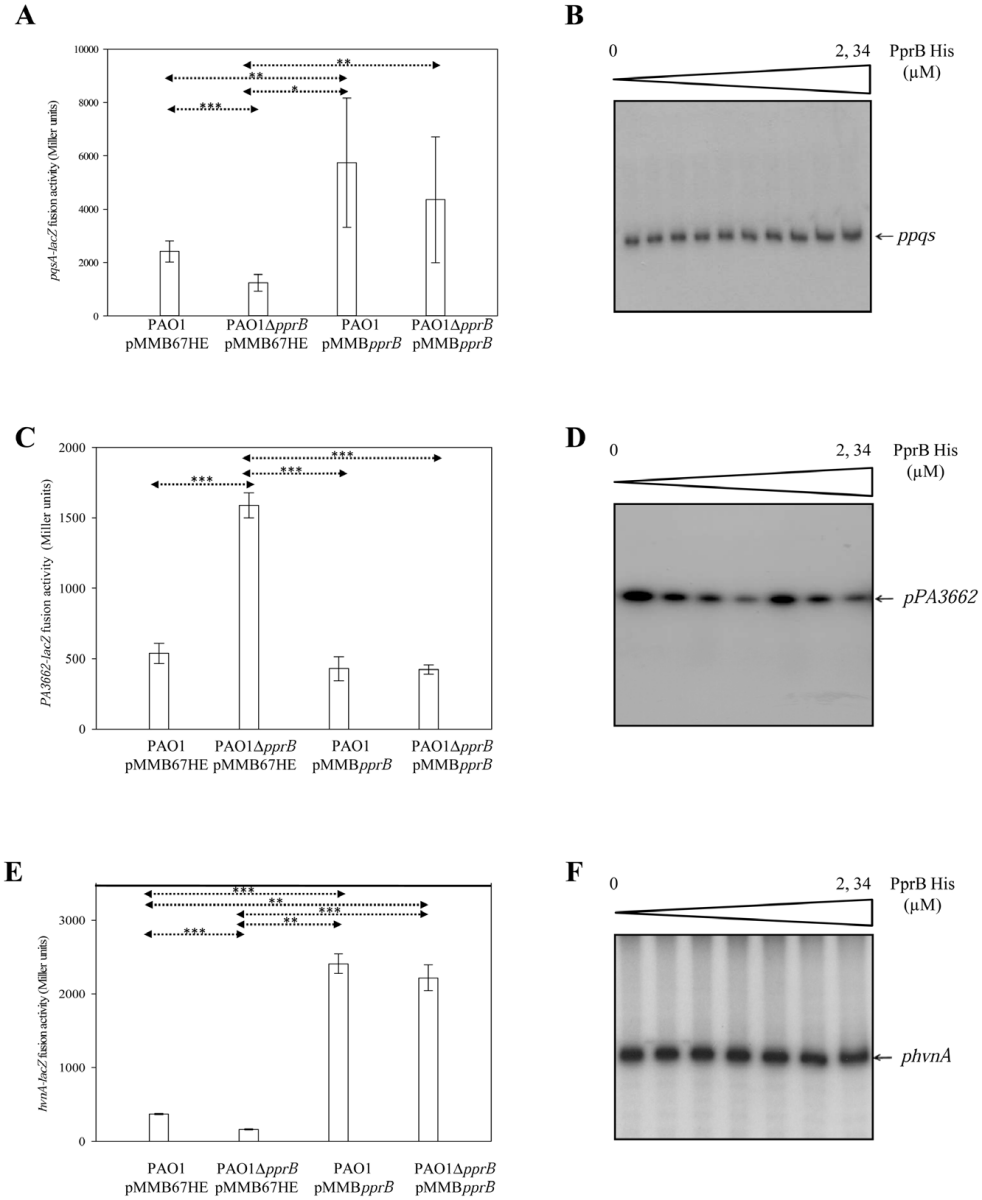
Validation of *pqs*, *PA3662* and *hvnA* genes. Expression of chromosomal *pqsA-lacZ* (**A**), *PA3662-lacZ* (**C**) and *hvnA-lacZ* (**E**) fusions was monitored in the PAO1/pMMB67HE, PAO1Δ*pprB*/pMMB67HE, PAO1/pMMB*pprB* and PAO1Δ*pprB*/pMMB*pprB* strains. Data are expressed in Miller units and correspond to mean values (with error bars) obtained from three or six independent experiments. Statistical analysis was performed using a Welch (C and E) or a t-test (A) (*:<0.05, **:<0.01, ***:<0.001). EMSAs performed with the purified PprB-6His protein, at concentrations of 0 to 2.34 µM, and the putative *pqs* (**B**), *PA3662* (**D**), and *hvnA* (**F**) promoter DNA regions.

*pqsABCDE*, *phnAB*, *PA1221-PA1216*, *PA1215-PA1211* loci, *PA3662* and *amtB* genes found to be differentially regulated in transcriptomic experiments were further analyzed. The corresponding chromosomal transcriptional fusions (*pqsA-lacZ*, *phnA-lacZ*, *PA1215-lacZ*, *PA1221-lacZ* and *gnlK-lacZ*) were introduced into the PAO1 wild type strain (carrying pMMB67HE or pMMB*pprB* vectors). Additionally, the *pqsA-lacZ* and the *PA3662-lacZ* fusions were introduced into the *pprB* mutant PAO1Δ*pprB* (carrying pMMB67HE or pMMB*pprB*). The *pqsA-lacZ* fusion was moderately but significantly induced in *pprB*-overexpressing conditions and down-regulated in the *pprB* mutant (Fig. 1A). An eight-fold increase in the levels of *pqsA* transcripts upon PprB overproduction (data not shown) observed by RT-qPCR fully confirmed this PprB-dependent effect on *pqs* gene expression. The activity of the *PA3662-lacZ* fusion was found to be significantly derepressed in the *pprB* mutant as compared to the wild type strain (Fig. 1C) and restored to the wild-type strain level when the *pprB* gene was brought *in trans*, fully confirming that this gene is under the negative control of the RR PprB. Using EMSA with a functional PprB-6His protein (see supplemental experimental procedures and Fig. S7), it appeared that the PprB-dependent control of *PA3662* and *pqs* genes was indirect (Fig. 1B and 1D), whereas in the same conditions, PprB-6His is able to bind to the *cupE* promoter (data not shown). None of the other fusions was responsive to *pprB* overexpression as illustrated for *phnA-lacZ* (Fig. S2A), *PA1215-lacZ* (Fig. S2B), *PA1221-lacZ* (Fig. S2C) and *glnK-lacZ* (Fig. S2D) fusions. We thus confirmed that the TCS PprAB exerts an indirect and positive while moderate but significant control on the *pqs* biosynthetic operon and an indirect but negative control on the *PA3662* gene.

### Halovibrin-like protein

Among the targets positively regulated by the TCS PprAB, the gene *PA1914*, renamed *hvnA* for Halovibrin A (see latter), was one of the most activated genes with a ≈30-fold induction. The *hvnA-lacZ* fusion although exhibiting a significant level in the wild type strain, was further induced in *pprB*-overexpressing conditions and down-regulated in the *pprB* mutant (Fig. 1E). Using EMSA, the DNA region of 630 bp encompassing the *hvnA* putative promoter was not retarded in the presence of the PprB-6His protein (Fig. 1F). Thus, the TCS PprAB positively but indirectly controls expression of the *hvnA* gene. The *hvnA* gene encodes a putative protein sharing 44% of identity with the two HvnA and HvnB proteins from *Vibrio fischeri* (Fig. S3A). It contains a putative NADase domain (an indirect ADP-ribosyltransferase (ARTase) activity leading to free ADP-ribose). However, in contrast to T3SS effectors possessing an ARTase activity that contribute to *Drosophila* fast killing [26], HvnA was not required for full virulence in *Drosophila* acute model of infection in the PAO1 genetic background (Fig. S3B) or in the PprBK genetic background (data not shown).

### A new large cell surface and secreted adhesion

**Figure 2:**
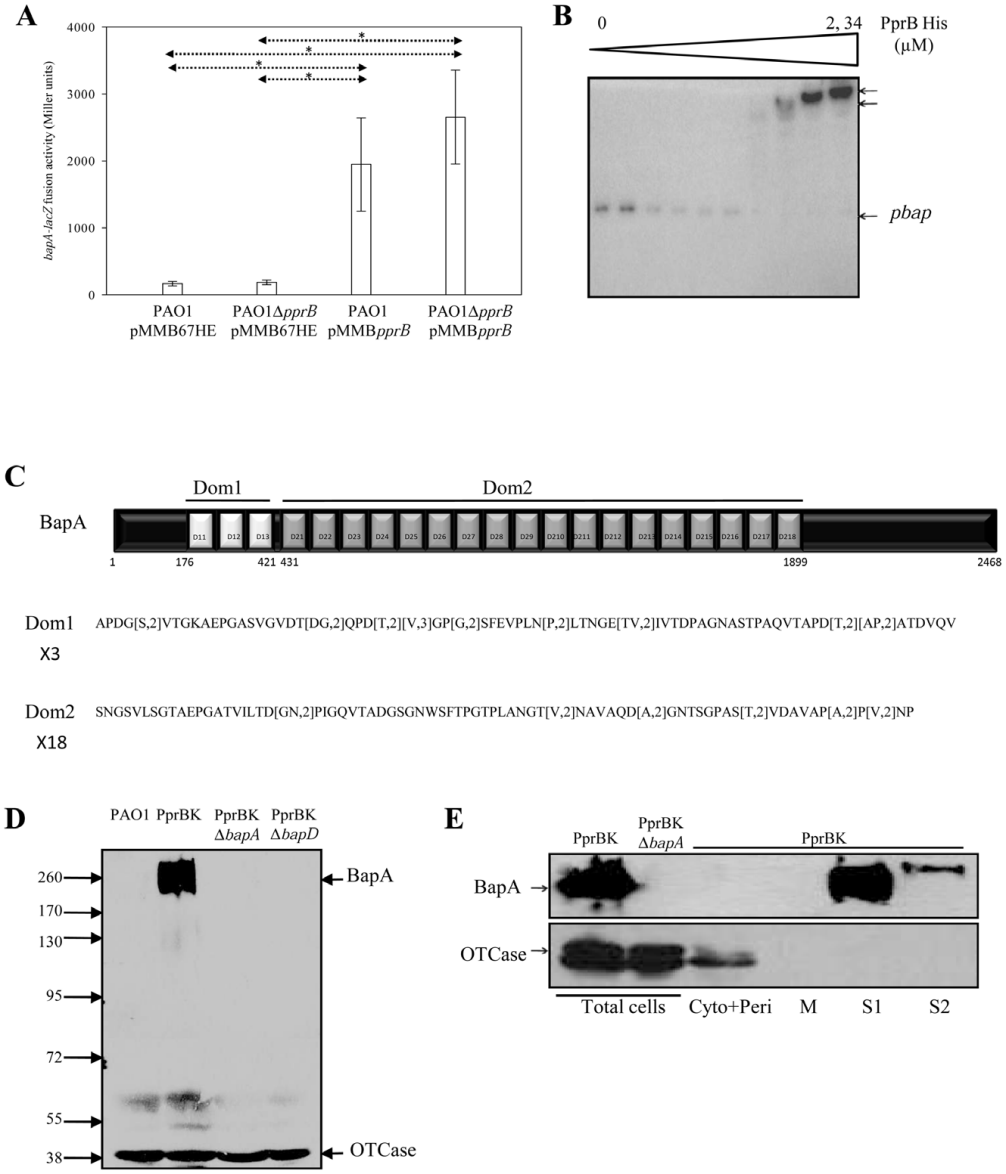
Validation of *bap* genes. Expression of the chromosomal *bapA-lacZ* fusion (**A**) was monitored in the PAO1/pMMB67HE, PAO1Δ*pprB*/pMMB67HE, PAO1/pMMB*pprB* and PAO1Δ*pprB*/pMMB*pprB* strains. Data are expressed in Miller units and correspond to mean values (with error bars) obtained from three independent experiments. Statistical analysis was performed using a Welch test (*:<0.05). EMSA (**B**) performed with the purified PprB-6His protein, at concentrations of 0 to 2.34 µM, and the putative *bap* promoter DNA region. Two retarded complexes (upper arrows) were identified at high PprB-6His concentrations. BapA protein from *P. aeruginosa* is a 2468 aa polypeptide (**C**) organized into two domains of 3 (Domain 1) and 18 (Domain 2) repeats of 82 and 86 aa, respectively. BapA production was detected (**D**) in western blot in the total cell extracts obtained from PAO1, PprBK, PprBKΔ*bapA*, and PprBKΔ*bapD* strains. As a control of equivalent loading between strains, OTCase protein was detected in the same samples. Number on left side is molecular weight standard (kDa). BapA localization (**E**) was checked in soluble (cytoplasmic and periplasmic fractions), in membrane (M) fractions, in classical (S1) and associated loosely with the cell surface (S2) supernatants. Cell leakage was specifically detected using anti-OTCase antibody.

Among the common targets identified in the two sets of microarray experiments is also the *PA1874-1877* gene locus. This locus of 4 genes forms a putative operon. Using appropriate overlapping oligonucleotides, we revealed that the four genes are transcribed as a polycistronic mRNA (Fig. S4A). Due to our findings (see latter), these genes in *P. aeruginosa* have been renamed, *bapA, bapB, bapC and bapD* for *PA1874*, *PA1875*, *PA1876* and *PA1877*, respectively. The *bapA-lacZ* fusion displayed a strict PprB-dependent expression (Fig. 2A) since it exhibited a rather null activity in the *pprB* mutant as well as in the wild type strain and *bapA* gene expression was significantly induced by the overproduction of the RR PprB, confirming the positive PprB regulation observed in microarray experiments. Using EMSA, the DNA region of 329 bp encompassing the putative promoter of *bap* genes formed at least two retarded complexes with PprB-6His concentrations higher than 0.34 µM (Fig. 2B). Thus, the TCS PprAB positively and directly controls expression of the *bap* gene locus.

The *P. aeruginosa* putative BapA protein for biofilm-associated protein A (2464 aa) is characterized by the absence of any peptide signal and by a two-domain organization (Fig. 2C), reminiscent of the LapA proteins of *P. fluorescens* WCS365 [30] and of *P. putida* KT2440 [31], of the LapF protein of *P. putida* KT2440 [32] and of the BapA protein of *S. enterica* [33]. The BapA protein of PAO1 strain consists of two domains, Domain 1of 3 and Domain 2 of 18 repeats of 82 and 86 aa, respectively (Fig. 2C). A lot of sequence variations could be observed in the corresponding putative proteins in the different *P. aeruginosa* genomes examined (see supplemental informations, Fig. S4B). The other *P. aeruginosa* putative Bap proteins are predicted to be an ATP-binding cassette (ABC) transporter formed of an ATPase of 723 aa (PA1876 or BapC protein), a membrane fusion protein of 395 aa (PA1877 or BapD protein) and an outer membrane (OM) protein of 425 aa (PA1875 or BapB protein). This transporter has been shown to participate in a biofilm-specific resistance mechanism involving efflux of drugs including tobramycin but also linked to *ndvB*-derived glucan mechanism [28]. Thus, this system unequivocally forms a T1SS transporter, potentially responsible for the export of the associated large protein BapA, a new member of large externalized, repeat-rich proteins emerging as important factors in the attachment of bacteria to biotic and abiotic surfaces [34], [35], that could be predicted to function as an adhesin. We therefore investigated the localization of BapA and its BapBCD-dependent secretion. Since no BapA production was observable in the PAO1 genetic background (Fig. 2D), further experiments were conducted in the PprBK genetic background. In this strain, we detected a strong production of BapA in total cell extracts, which migrates at an apparent molecular weight around 260 kDa (Fig. 2D), a specific signal that was further assessed by the absence of BapA protein in the *bapA* mutant. BapA was not detected in the same samples derived from the *bapD* mutant (Fig. 2D), suggesting that in the absence of one element of the transporter, here BapD, the substrate BapA is unstable, and that BapD could participate to BapA stabilization. We further checked where it localized. BapA was not associated with membranes (M), but recovered in classical supernatant (S1) as well as associated loosely with the cell surface and therefore can be removed from the OM using quick and gentle vortexing procedure, since it was also recovered in the second supernatant (S2) (Fig. 2E).

### PprB dependent phenotypes

To define a general view of PprBK strain behavior, biofilm formation in flow cell, drug susceptibility, cytotoxicity toward eukaryotic cell lines (HeLa and J774) and virulence in flies were examined.

**Figure 3:**
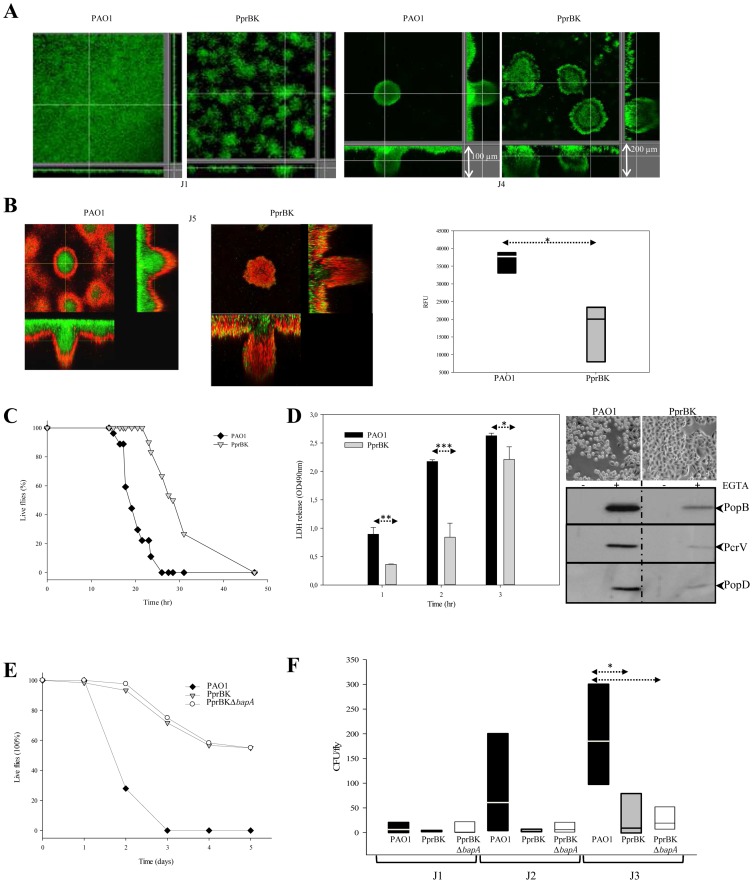
PprB-dependent phenotypes. Biofilm formation monitored at day 1 (J1) and 4 (J4) of PAO1_GFP_ and PprBK_GFP_ strains (**A**). Antimicrobial tolerance of PAO1 _GFP_ and PprBK_GFP_ -5day aged biofilms exposed to 20 µg/ml of tobramycin for 24 hr (**B**). Dead cells were labeled with propidium iodide. Extracted *z* images and their respective *xy* and *xz* planes are presented. Resazurin viability test was performed in PAO1 and PprBK biofilms formed in microplates and exposed to 20 µg/ml of tobramycin for 24 hr. Relative fluorescence (RFU) was measured using λ_exc_ and λ_em_ of 530 nm and 595 nm, respectively. Independent measurements were submitted to a one way statistical test (*:<0.05). PprBK strain virulence was examined in *D. melanogaster* model (**C**) using fast killing assay and compared to PAO1. Survival curves (% of live flies according to time of infection) were drawn and each point represents a total of 30 flies displayed in 3 different vials by group of 10. One representative experiment out of three is shown. PprBK strain virulence (**D**) was tested in the HeLa cell model (right and upper panel) and compared to the PAO1 strain. Rounding of cells was surveyed under optical microscopic observation. Cytotoxicity of PAO1 and PprBK strains towards J774 murine macrophages was evaluated by LDH release measured at OD_490_ (Left panel). Statistical differences were evaluated using a t-test (*:<0.05, **:<0.0.01, ***:<0.001). *In vitro* secretion of T3SS proteins forming the translocon (PopB, PopD and PcrV) was checked with (+) or without (−) inducing conditions (EGTA) in PprBK and PAO1 strains (right and lower panel). Bacterial virulence was checked in *D. melanogaster* oral infection assay (**E**) for PprBK, PprBKΔ*bapA* and PAO1 strains. Survival curves (% of live flies according to time of infection) were drawn and each point represents a total of 60 flies. One representative experiment out of three is shown. Dissemination of bacteria from the gut lumen toward the hemolymph compartment (**F**) was evaluated for PprBK, PprBKΔ*bapA* and PAO1 strains by counting CFU in hemolymph collected and pooled from 10 infected *Drosophila* per strain. Box plots represent the data obtained on 3 independent experiments made for each strain. Statistical differences were evaluated using a Kruskal-Wallis One Way Analysis (*:<0.05).

The PprBK strain displayed a peculiar hyper-biofilm phenotype, allowing cells to gather in microcolonies as soon as day one (J1) of biofilm development with significant three dimensional expansion while at the same time point the PAO1 strain formed a single layered-cell carpet on the glass substrate (Fig. 3A). This was indeed much more observable after four days (J4), where the PprBK strain was forming three dimensional mushrooms two-fold higher than the PAO1 strain (roughly 200 µm for the PprBK as compared to 80 µm for the PAO1 strain). Unexpectedly, PprBK was two-fold more susceptible to tobramycin in particular under biofilm growth conditions as compared to PAO1 strain in a very reproducible manner (Table S3). Concordant with these results, the number of dead cells observed was strongly increased in the PprBK biofilms as compared to the PAO1 biofilms after exposure to tobramycin (Fig. 3B). Whereas centers of mushrooms contained live bacteria in the PAO1 formed biofilm, they were always containing a majority of dead cells in the PprBK one. Additionally, whereas susceptibility to tobramycin was restricted to the superficial layer of the PAO1 - formed biofilms, it has reached in depth cells of the PprBK formed biofilm (Fig. 3B, left panel). This result was confirmed by the quantitative resazurin test in which PprBK - formed biofilms exhibited a significantly reduced viability as compared to PAO1 - formed biofilms (Fig. 3B, right panel).

Characterization of the PprB-dependent virulence was then assayed in *D. melanogaster* and compared to the one of the PAO1 strain. When introduced in flies by septic injury to produce acute infection, the PprBK strain was found to be strongly attenuated in virulence (Fig. 3C), with LD_50_ reached after 18 hr and 28 hr of infection, in PAO1 and PprBK strains, respectively. Since, fast *Drosophila* killing has been mainly associated with T3SS activity [5], [26], we then compared the capacity of the PprBK strain to induce cytotoxicity on cultured epithelial HeLa cells and murine macrophage J774 cell line. In both assays, compared to the PAO1 strain, PprBK was clearly found less cytotoxic with delayed effects on LDH release measured for infected macrophages and cell rounding of HeLa cells (Fig. 3D, left panel; upper and right panel). In concordance with cellular effects, PprBK secreted significantly less T3SS proteins, namely PcrV, PopB and PopD in culture supernatants (Fig. 3D, right and lower panel), further arguing that reduced virulence of the PprBK strain in acutely infected *Drosophila* can be attributed to reduced T3SS activity.

*Drosophila* can also be orally infected mimicking a chronic model of infection. The hyper-biofilm *P. aeruginosa* strain (PAZH) has been recently reported to produce reduced virulence in both acutely and chronically infected flies [24]. Interestingly, the PprBK strain was also found to be strongly attenuated in virulence in orally infected flies (Fig. 3E), as compared to the PAO1 strain. Moreover, the PAO1 strain was detected in the hemolymph two days post infection and the total average amount reached ≈200 CFU/fly three days post infection, while PprBK strain was barely detected in the hemolymph at day 3 with repeatedly less than 50 CFU/fly collected (Fig. 3F, p = 0.041). Deletion of *bapA* gene did not alter virulence as compared to its isogenic parental strain PprBK (Fig. 3E) and did not restore the capacity of the PprBK strain to cross the digestive barrier (Fig. 3F). These results indicate that decreased PprB-dependent virulence is tightly associated with dissemination defect through the *Drosophila* digestive barrier. This suggests that secreted molecules produced in PprBK other than BapA may prevent dissemination, and/or that products of some genes repressed in the PprBK context are required to cross the epithelial barrier.

Taken together, these results are in favor with a TCS PprAB dependent hyper-biofilm phenotype associated with decreased virulence in acute and chronic cell or animal models and drug hyper-susceptibility.

### BapA, Type IVb pili, CU fimbriae and eDNA contribute to the PprB-dependent biofilm phenotype

**Figure 4:**
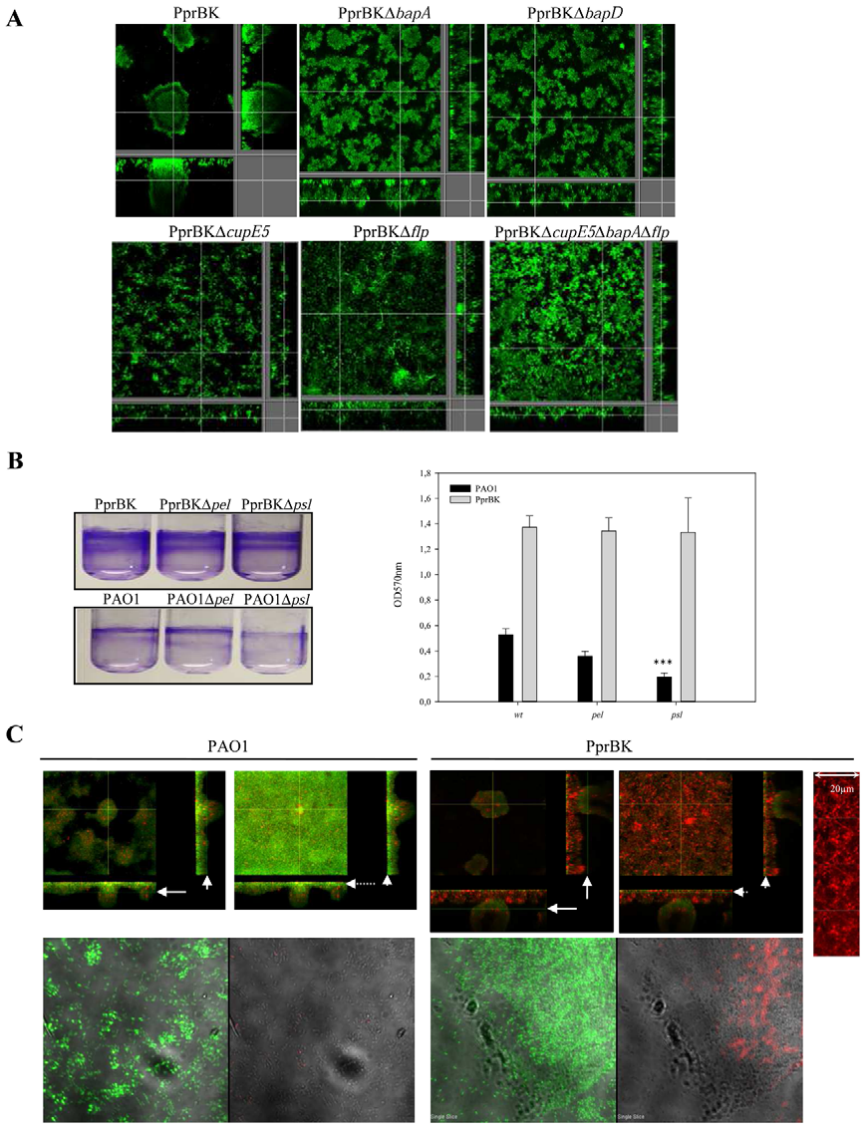
Contribution of PprB-dependent biofilm matrix in biofilm structuration. Biofilm formation monitored under dynamic conditions (**A**) at day 4 of PprBK_GFP_, PprBKΔ*bapA*_GFP_, PprBKΔ*bapD*_GFP_, PprBKΔ*cupE5*_GFP_, PprBKΔ*flp*_GFP_, and PprBKΔ*cupE5*ΔbapAΔflp_GFP_ strains. Crystal violet (CV) - stained biofilms (**B**) formed by PprBK (wt), PprBKΔ*pel* (*pel* mutant), PprBKΔ*psl* (*psl* mutant), PAO1 (wt), PAO1Δ*pel* (*pel* mutant) and PAO1Δ*psl* (*pel* mutant) strains (tube views, left panel). CV staining was extracted and quantitative analysis was performed. Data presented represent means values and standard deviations obtained at least 4 independent experiments (right panel). Upper panel: biofilm formation monitored under dynamic conditions (**C**) at day 4 of PAO1_GFP_ and PprBK_GFP_ strains in which eDNA was revealed using propidium iodide (red labeling) in intermediate (plain arrows) (left images) and low (dotted arrows) projections (right images). Extracted *z* images and their respective *xy* and *xz* planes are presented. Enlarged view of the meshing formed by the eDNA present at the base of the PprBK biofilm. Lower panel: biofilm formation monitored under static conditions (**C**) at day 4 of PAO1_GFP_ and PprBK_GFP_ strains in which eDNA was revealed using DDAO probe (red labeling). Combined transmitted and green (left images) and combined transmitted and red (left images) images were shown.

We further examined whether BapA could be a potential new adhesin playing a role in *P. aeruginosa* biofilm formation. The single mutants PprBKΔ*bapA* and PprBKΔ*bapD* displayed an altered biofilm phenotype as compared to the isogenic parental strain PprBK (Fig. 4A), thus confirming that BapA is strongly involved in the PprBK hyper-biofilm phenotype and that BapA is an adhesin exported by the T1SS BapBCD transporter. Additionally, PprBKΔ*cupE5* and PprBKΔ*flp* mutants displayed altered mushroom structures (Fig. 4A) in a rather identical manner than did the PprBKΔ*bapA* mutant, with poorly packed microcolonies that had not expanded in 3D and were not forming mushroom-shaped structures. Additional triple mutation was generated in this strain, thus leading to the PprBKΔ*cupE5*Δ*bapA*Δ*flp* strain, which displayed a very similar phenotype to any of the single mutants (Fig. 4A), suggesting that CupE CU fimbriae, Flp Type IVb pili, and BapA adhesin all cooperate in the PprBK hyper-biofilm phenotype and that absence of any of them strongly destabilizes the biofilm formed. Since no particular EPS genes (*pel*, *psl*, *alg*) have been identified as controlled by the TCS PprAB (Tables S1 and S2) and additionally confirmed by RT-qPCR (data not shown), we investigated whether any of the exopolysaccharides Pel or Psl associated with non mucoid phenotype contribute to the PprBK hyper-biofilm phenotype, PprBK having a non mucoid phenotype on plate. In frame deletion *pel* and *psl* mutants were engineered in the PAO1 and PprBK genetic backgrounds and examined as well. It appears that whilst Psl mainly impacts PAO1 biofilm (Fig. 4B, p<0.001), Pel has a non significant impact as described earlier [36]. The hyper biofilm of PprBK strain was unmodified in the corresponding *pel* and *psl* mutants as compared to their isogenic parental strain PprBK, strongly reinforcing the idea that once the PprBK signaling pathway is activated, the hyper biofilm observed does not rely on Pel and Psl exopolysaccharides (Fig. 4B illustrates the different crystal violet stained biofilms and corresponding quantitative analysis). Since we observed a significant induction of *pqs* genes that have been previously reported to control exogeneous DNA release (eDNA) [37], we further looked at the presence of eDNA in the observed PprBK-dependent hyper-biofilm. Indeed, eDNA was substantially present in the PprBK biofilm as compared to the PAO1 one, in particular in lower layers (Fig. 4C, upper panel), forming meshing (Fig.4C, upper panel, enlarged view). A DDAO staining was additionally performed, this probe being recognized for its affinity for double stranded DNA, good fluorescence properties, molecular size preventing it from penetrating intact membranes and therefore targeting only eDNA [37], [38]. Bacterial aggregates within the PprBK formed - biofilms displayed a positive DDAO staining while those of PAO1 formed – biofilms were slightly stained (Fig. 4C, lower panel). Taken together, these results highly suggest that eDNA release plays a very important role in shaping the PprBK hyper-biofilm phenotype, whilst at the same time Psl or Pel EPS are useless.

**Figure 5:**
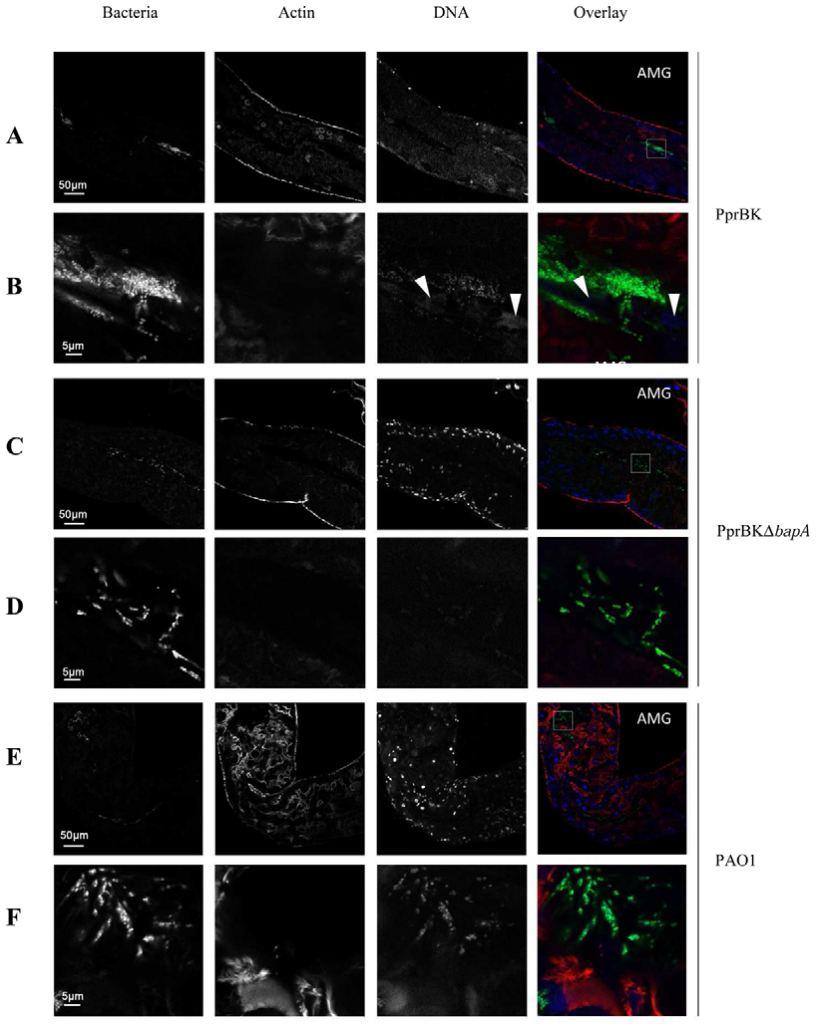
Confocal sections of *Drosophila* anterior midguts (AMG). Flies were orally infected with GFP expressing bacteria (green); guts were dissected at one day post-infection and stained with Alexa 546-phalloidin (actin, red) and DAPI (DNA, blue). Sections of PprBK_GFP_ (**A**), PprBKΔ*bapA*_GFP_ (**C**) and PAO1_GFP_ (**E**) infected anterior midguts. Magnifications of corresponding indicated squares (PprBK_GFP_ (**B**), PprBKΔ*bapA*_GFP_ (**D**) and PAO1_GFP_ (**F**)). Particular high density area of PprBK_GFP_ bacteria surrounded with eDNA (arrowheads) (**B**) was shown.

To further assess the capacity of PprBK to form biofilms *in vivo* and the contribution of BapA in this process, flies were orally infected with PAO1, PprBK and PprBKΔ*bapA* strains. In all three cases, one day following infection, large amounts of bacteria were found in the crop lumen (shown for PAO1 in Fig. S5) and in the proventriculus (PV) (Fig. S6). Additionally, PprBK infected guts displayed dense bacterial aggregates located along the *Drosophila* epithelium in the anterior midgut (AMG) (Fig. 5A and B) mostly surrounded with extracellular DNA (see arrowheads in Fig. 5B) and which are likely to correspond to *in vivo* biofilms. In contrast, bacteria were found mostly dispersed in PprBKΔ*bapA* (Fig. 5C and D) and PAO1 infected flies (Fig. 5E and F). PprBK and PAO1 cells were found in close contact with the intestinal barrier (Fig. S6C and Fig. S5C respectively). Of note, bacterial aggregates were observed not only in the crop as previously reported [24], but also in various parts of the intestinal tract such as the anterior midgut (AMG) (Fig. 5) and the proventriculus (PV) (Fig. S6). Although, sometimes massively present in the lumen gut (not shown), the PprBKΔ*bapA* strain was found to be strongly impaired in its capability to either stick to *Drosophila* epithelial tissues or to disseminate towards the hemolymph compartment (Fig. 3F). Instead, PprBKΔ*bapA* bacteria were taking part to a long sticky viscous filament observed upon dissection probably cemented by extracellular material and/or adhesive properties of bacterial cells themselves (data not shown).

Taken together, these observations indicate that PprBK forms biofilm on the mucosal surface in the midgut compartment a phenomenon requiring the BapA adhesin, while it is impeded in dissemination independently of BapA.

## Discussion

In the present study we unraveled the genes belonging to the *P. aeruginosa* TCS PprAB regulon among which we identified a particular association of molecular actors involved in biofilm formation. We identified BapA, a large cell surface and T1SS secreted protein. BapA is the first member in *P. aeruginosa* of a family of large proteins involved in adhesion and biofilm formation extensively growing in Gram positive and Gram negative bacteria. They all exhibit substantial sequence identity with expansive domains with imperfect tandem repeats of 86–106 amino acids, and are strikingly large, cell surface-localized and/or secreted proteins. In Gram–negative bacteria, they all share a T1SS mechanism, through a transporter possessing ABC characteristics generally closely associated in an operon together with their respective substrates. Evidence has been provided that this kind of secreted proteins promotes multicellular cell-to-cell interactions, however whether they form filamentous structures and presumptive calcium-binding domains influence the adhesive and cohesive processes remain to be answered [35]. BapA from *P. aeruginosa* is indeed secreted by the T1SS transporter encoded by the adjacent *bapBCD* genes and possesses the GGDGSD T1SS sequence consistent with the observed instability of the substrate in the absence of its cognate BapBCD transporter. This sustained a strong coupling between BapA synthesis and BapBCD-dependent secretion, as previously reported [39]. BapA forms with CupE fimbriae from the CU family and Type IVb pili, a molecular toolkit in which all cooperate to shape biofilm development as it has been reported for BapA in *S. enteritica* with cellulose and curli fimbriae upon the common regulation of *csgD*. CsgD – dependent direct and positive expression of *csgBA* and *adrA* genes encoding cellulose and curli fimbriae [40] has been coupled to *bapA* gene induction [33], [34] in *S. typhimurium*. Thus, as for CsgD in *S. typhimurium*, the PprB regulator may represent a checkpoint of multicellular and aggregative behavior in *P. aeruginosa* [41]. This renders BapA protein from *P. aeruginosa* closer to BapA from *S. typhimurium* rather than to Lap proteins of the pseudomonads. Lap protein regulation has been described to be controlled by c-di-GMP intracellular messenger through proteins encoded by genes close to their respective targets, such as LapD for LapA, a protein with degenerate GGDEF and EAL motifs [42] and probably PP_0798 encoded protein, a GGDEF-type protein for LapF. There is a gene encoding a GGDEF protein PA1851 in the vicinity of *P. aeruginosa bapA* gene (*PA1874*). However, its overexpression or deletion did not lead to a particular biofilm phenotype in the PA14 strain, suggesting that it is probably not involved in controlling *bapA* regulation in *P. aeruginosa* [43]. We cannot exclude that it may occur in the PAO1 genetic background or in the PprB activated context, even though this particular gene is not regulated by PprB. Whether *P. aeruginosa* BapA acts through homophilic interactions such as the *S. enteritica* BapA protein [33], or whether, it participates to physical association with CupE fimbriae or/and Type IVb pili in the biofilm as it has been reported for the *P. aeruginosa* CdrA protein (a two partner secretion or Type Vb large substrate) with Psl extracellular polysaccharide matrix [44] remains to be elucidated. Interestingly, BapA adhesin appears to be a major determinant in biofilm establishment and attachment on the mucosal surface of the midgut observed in orally infected *Drosophila*. Indeed PprBΔ*bapA* was incapable to stick to *Drosophila* epithelial tissues and was strictly restricted to the lumen of the gut while it still possesses an aggregative behavior in various gut compartments (crop, proventriculus). This contrasts with the strong attenuation of *in vitro* biofilm in the *bapA* mutant as compared to the PprBK parental isogenic strain, suggesting that there is probably beside the bacterial produced matrix, *Drosophila* material that could also interfere with the remaining bacterial cell structures, in particular Type IVb pili and/or CupE CU fimbriae.

In this particular extracellular matrix produced upon the PprB activation, eDNA release through probable PQS regulatory pathway activation is also playing a key role in shaping the community and therefore contributes to multi-cellular development processes as previously reported [37]. DNA cement was also observed in the chronic *Drosophila* model used here. The involvement of PQS in DNA release [37] has been reported earlier and strongly contributes to the formation of huge mushroom-shaped structures [45]. *pqsA* expression specifically occurs in microcolonies in the early phases of biofilm formation [37] and reaches a maximum level in 2-day-old biofilms [45]. Moreover this particular time and location expression is critical for cap formation [45]. Here, eDNA that mainly localized in cell layers in contact with the substratum, is thus a cell-to-cell interconnecting matrix component in PprBK biofilm that contributes together with Type IVb pili, fimbriae of the CU pathway and BapA protein to the hyper-biofilm phenotype of this strain. From the present study, we highlighted a hyper-biofilm phenotype without major involvement of EPSs as undoubtedly proved with unaltered hyper-biofilm phenotype of the *pel* and *psl* mutants in the PprBK genetic background such as those previously reported in *P. aeruginosa*. This very interesting and new feature has been already observed for Rhizobium which is able to form biofilms on roots of non-legumes, independently of EPS synthesis [46], thus reinforcing the notion that EPS is not an absolute marker of biofilm lifestyle.

Beside this hyper-biofilm phenotype associated with PprAB activation, we observed a decreased T3SS secretion in the PprBK strain as compared to the PAO1 strain associated with a reduced cytotoxicity in epithelial and macrophage lineages. This matches with the idea that biofilm is regulated in an opposite way from cytotoxic-mediated and T3SS-dependent virulence as demonstrated for many pathways in particular those involving small regulatory RNAs RsmY and RsmZ [47]. This probably occurs through PQS level, which high level is negatively influencing T3SS effector secretion [48]. Decreased T3SS expression (Table S2) may also account for decreased virulence in acute *Drosophila* model of infection in which T3SS is known to play a major function [5]. Interestingly, our *P. aeruginosa* hyper-biofilm strain showed reduced virulence in both acute and chronic *Drosophila* infection similarly to what was described for the hyper-biofilm PAZH strain [24]. As previously observed in the case of PAZH (Mulcahy et al., 2011), decreased virulence of PprBK is tightly associated with its incapability to cross the intestinal barrier and to reach the hemolymph. However, in contrast to PAZH, biofilms formed by PprBK were found not only in the crop but also in various parts of the intestinal tract including the proventriculus and the midgut.

In the present study, we also unraveled the PprB-dependent activation of the *hvnA* gene encoding a putative ADP-ribosylating toxin that has never been identified so far in this bacterium. No HvnA-dependent virulence phenotype could be observed in a first round of assays, suggesting that either HvnA targets other host proteins than do ExoS and ExoT ARTases, or that host proteins targeted by this protein are not present in the *D. melanogaster* model. Even if HvnA is translated (detected in bacterial crude extracts, data not shown), there is no evidence yet of its secretion. HvnA displays a high identity in its N-terminal domain with the halovibrin from *V. fischeri* which has been initially thought to catalyze polyarginine ribosylation using NAD1 as a substrate [49], but also polylysine and polyhistidine ADP-ribosylation thus releasing free, reactive, ADP-ribose through a NAD+-glycohydrolase activity (NADase) [29]. Clinical streptococcal isolates and *V. cholerae* secrete NADases [50], [51], which observation combined with our results, may initiate further study of the role of secreted NADases in bacterium-host interactions.

An original observation made in the present study is the association of a hyper-biofilm phenotype with an increased susceptibility to tobramycin, whereas biofilm lifestyle has been extensively associated with resistance to antibiotics. In contrast with a previous study [28], suppression of BapBCD transporter showed no influence on tobramycin susceptibility (Table S3). Our results would thus indicate that the increased susceptibility to tobramycin in biofilm is probably due to the particular nature of the matrix exopolymer sealing the community and/or to the increased membrane permeability linked to PprB [20]. Drug susceptibility could also be explained by the PprB-dependent repression of the *mexXY* operon that encodes part of the tripartite multidrug efflux pump MexXY-OprM (Table S1). This hypothesis was ruled out by the absence of PprB-dependent change in the level of *mexXY* mRNA assessed by RT-qPCR (data not shown), as well as by the observation of equal drug resistance of a *mexXY* mutant under activated - or inactivated - PprB pathway conditions. This later experiment further ruled out other mechanisms and in particular those implicating ribosomal targets. Additionally and very interestingly, an increased susceptibility to aminosides has been linked to elevated intracellular polyamine levels. In *E. coli*, the ammonium transporter AmtB is thought to import ammonia and to lead to increased intracellular level of polyamines [52], these latter having been shown to increase translation of OppA, a periplasmic binding protein involved in aminoglycoside uptake [53]. This was a very seductive aspect to mention since in our study, the *amtB* gene has been shown to be differentially regulated in our transcriptomic study. While the *glnK-amtB* operon was potentially interesting for further drug susceptibility phenotype interpretation, we were unable to observe any change in *amtB* transcription activity or quantities of *amtB* mRNA upon PprB activation by RT-qPCR. Thus the particular matrix surrounding PprB-activated biofilm could be the major determinant of associated increased susceptibility to tobramycin, a hypothesis which would deserve further investigation.

Finally, this work demonstrates that the TCS PprAB in *P. aeruginosa* may represent a key bacterial adaptation checkpoint of multicellular and aggregative behavior combining a particular adhesive signature, associating increased drug susceptibility and decreased virulence, a particular interesting therapeutic window to consider in eradication of *P. aeruginosa* biofilm-associated infections. Even though we studied a particular regulatory pathway (artificially activated in PprBK strain), recent data were obtained in early CF strains that showed that this PprB pathway is activated in humans [54] and reinforcing the idea that there could be timing where drugs could be more efficient even in biofilm-associated infections.

## Materials and Methods

### Microbial genetic procedures

Bacterial strains, growth conditions, deletion mutants and chromosomal fusions are described in the supplemental experimental procedures (Tables S4, S5, S6). The expression of the different transcriptional fusions was monitored by assaying ß-galactosidase activity as previously described [23].

### EMSAs

Electrophoretic mobility shift assays (EMSAs) were performed as follows. 329 bp, 630 bp, 612 bp, 348 bp DNA regions of the *bapA, hvnA, pqsA, PA3662* putative promoters were amplified by PCR using the appropriate oligonucleotide pairs (Table S6) in the presence of αP^32^ dGTP. A mixture of each PCR product (50 ng) and of sonicated salmon sperm DNA (2 µg/µl, ratio 1∶40 of the different promoters and the competitor DNA) in a 50 mM Tris-HCl buffer pH 8.2 supplemented with 1 mM EDTA and 0.25 mM saccharose, was incubated for 30 min at room temperature with various concentrations of purified PprB-6His protein ranging from 0 to 2.34 µM. Samples were resolved on a pre-run 12%-acrylamide gel in Tris-borate buffer. Gels were fixed in 10% trichloro-acetic acid for 10 min, and exposed to Kodak BioMax MR films.

### Cell fractionation and supernatant preparation

Bacterial cells were harvested, resuspended in Tris HCl 10 mM pH 8.0 with protease inhibitor cocktail, and disrupted. To remove unbroken cells additional centrifugation at 5,000 rpm was done before ultracentifugation for 30 min at 45,000 rpm. Upper soluble phase fraction contains both cytoplasmic and periplasmic fractions, whereas the pellet contains membranes. Supernatants S1 and S2 were collected as previously described [30]. Cell extracts and supernatants were loaded at 0.1 OD_600_ unit for total cell extracts, 0.1 OD_600_ unit for soluble (cytoplasmic and periplasmic fractions) fractions, 0.5 OD_600_ unit for membrane (M) fractions, 1 OD_600_ unit for classical (S1) and associated loosely with the cell surface (S2) supernatants. Proteins were separated by electrophoresis in an 8% polyacrylamide gel. BapA protein was immunodetected with the polyclonal antibodies against BapA (1∶1,000, (produced as described in the supplemental experimental procedures) and leakage was followed by immunodetection of the cytoplasmic ornithine carbamoyltransferase (OTCase) protein using a specific antibody (a gift from Dieter Haas, Université de Lausanne, Switzerland) at a dilution of 1∶500.

### Biofilm formation

Time-lapse biofilm formation was performed in flow chambers as described previously [23]. The *P. aeruginosa* strains were tagged with green fluorescent protein (GFP), as described elsewhere [55]. Observation was performed with an Olympus FV-1000 microscope equipped with detectors and filter sets for monitoring of GFP, propidium iodide. The flow cell inoculation, the running of the system, the microscopic inspection, the image capture and the analysis were carried out as previously described [56]. The *pel* and *psl* mutants were grown in parallel to their isogenic parental strains under static conditions at 30°C and attached bacteria were stained with 1% Crystal Violet. Staining was extracted by treatment with 400 µl 95% ethanol. Subsequently, 600 µl of water was added and OD_570_ was measured. All quantification assays were made at least in triplicate. eDNA staining was performed using a combination of fluorescent probes including propidium iodide and DDAO as previously reported [38] and observed with appropriate filters.

### Transcriptional profiling using microarrays

Cells were grown in LB for 8 hr at 37°C in the presence of 10 µM of IPTG when strains carried plasmids. RNAs were prepared using the Midiprep Total RNA Isolation System (Promega). The integrity of RNA preparations was checked by the Experion automated electrophoresis system (Bio-Rad) and the absence of DNA contamination was verified by PCR. 500 ng of RNA were further processed for double Cy3/Cy5 labelling using the MessageAmp II aRNA Amplification Kit (Ambion). Dye incorporation rates were measured using a Nanodrop ND-1000 spectrophotometer (Nanodrop technologies Wilmington, DE). 300 ng of Cy3-labelled and of Cy-5 labelled aRNA mixtures were hybridized for 17 hr at 65°C according to Agilent protocols on the MGPA DNA Chips. MGPA chips and data analysis are described in supplemental experimental procedures.

### *Drosophila* acute and chronic infections

Flies infections were performed using diluted bacterial exponential phase cultures (0.5 OD_600 nm_) as described previously [5], [26], [57]. 30 and 60 *w^118^* (control genotype) males were infected in each experiment for septic and oral infections respectively and distributed ten by ten into vials. One experiment out of three is shown. Additionally, calibrated drops of hemolymph were sampled from 10 infected flies per bacterial strain, diluted in 100 µl of LB and serial dilutions were further plated on PIA. Colony-forming units (CFU) were numbered after overnight incubation at 37°C.

### *Drosophila* guts staining

Guts from infected *Drosophila* were dissected in PBS 24 hr following oral infection and fixed for 40 min in 4% paraformaldehyde. After twice rinsing in PBS, samples were incubated overnight at 4°C in 2 U/ml Alexa Fluor 546 Phalloidin (Invitrogen). After mounting in DAPI containing Vectashield (Vector Laboratories, H-1200), the samples were imaged with a 40× or 63× magnification (oil immersion) using a Leica TCS SP2 confocal microscope and the LCS software. A total of at least 5 dissected intestines per experiment which have been reproduced three times were performed.

### HeLa cell, J774 murine macrophage infection and T3SS secretion

T3SS-dependent cytotoxicity was evaluated on cultured epithelial HeLa cells and J774 murine macrophages [58]. Cytotoxicity was evaluated on J774 cells by measuring LDH release using Cytotoxicity Detection kit (Roch) after 1, 2 and 3 hr of contact with bacteria at a MOI of 10. Images of infected HeLa cells were obtained at 3 hr post-infection using Leica DM IRE2 microscope. Induction of T3SS *in vitro* was obtained by adding 5 mM EGTA and 20 mM MgCl_2_ to bacterial cultures at OD_600 nm_ of 0.1. After 3 hr of growth at 37°C with agitation, supernatants were collected and analyzed by immunoblot using anti-PcrV, anti-PopB, anti-PopD polyclonal antibodies [58].

### Antibiotic susceptibility

The minimal inhibitory concentrations (MICs) of tobramycin and ciprofloxacin were determined as recommended by the CLSI by the dilution method in Mueller Hinton agar [59]. The minimal bactericidal concentrations for biofilm (MBC-B) and planktonic cells (MBC-P) were determined as previously described [28]. Additionally, susceptibility towards tobramycin was further checked in flow cell experiments. At day 4, biofilms formed by PAO1 and PprBK strains in flow cell chambers were exposed to 20 µg/ml of tobramycin for 24 hr at 30°C. Dead cells were revealed by additional propidium iodide exposure for 10 min and resulting red cell populations were observed using appropriate filter.

## Supporting Information

Figure S1 Schematic diagram of the *flprcptad* and *cupE* loci and the six new loci gathering the 35 genes identified as differentially regulated by the RR PprB. The PprB-controlled genes identified in the microarray experiments are grayed. Numbers on the right side refer to the size of the corresponding DNA regions in kilobases (kb).
(TIF)

Figure S2 Expression of the chromosomal *hvnA-lacZ* (**A**) *PA1215-lacZ* (**B**), *PA1221-lacZ* (**C**), *glnK-lacZ* (**D**) fusions was monitored in the PAO1/pMMB67HE (black circles) and PAO1/pMMB*pprB* (open circles) strains grown for 8 hr in the presence of 10 µM IPTG. Data are expressed in Miller units and correspond to mean values (with error bars) obtained from three independent experiments. The corresponding growth curves (dotted curves) are presented.
(TIF)

Figure S3 HvnA protein from *P. aeruginosa* is 408 aa polypeptide (**A**) with a signal peptide (aa 1–24), a HvnA domain (aa 25–301) with an RGD sequence at position 174 and C-terminal DUF2599 domain (aa 302–408). ClustalW alignment of the halovibrin domain of HvnA from *P. aeruginosa* with HvnA of *V. fischeri* (regions I, II and III, the region III constituting the active site for NAD+ hydrolysis are squared and RGD sequence is underlined). PAO1 and PAO1Δ*hvnA* strains were examined for virulence in *D. melanogaster* model (**B**) using fast killing assay. Survival curves (% of live flies according to time of infection) were drawn and each point represents a total of 30 to 50 flies. One representative experiment out of three is shown.
(TIF)

Figure S4 Genetic organization (**A**, Upper panel) of the *P. aeruginosa bapABCD* locus. Above is the precise localization of primers used to target each contiguous gene junction (see Table S6) for determination of operonic structure along the locus. Corresponding bands (**A**, Lower panel) with expected size obtained after PCR on genomic DNA were compared to the ones obtained after RT-PCR on RNA (cDNA). Absence of DNA contamination of RNA was controlled. DNA ladder and sizes in bp are precised on the left. Analysis of BapA sequences (**B**) from PA14, LESB58, C3719, 39016 and PA7 strains performed using Xstream and compared to the BapA sequence from PAO1 strain. Deletions were observed in Domain 1 for 39016 (D12/D13) and PA7 (D12) strains. In Domain 2, insertions of repeats D216S1/D216S2/D216S3 in PA14 and LESB58 strains, D214S1 and D215S1/D215S2 in PA7 strain and deletions of D216 for C3719 strain and D24/D25/D26 for 39016 strain were observed. Polymorphism raises 22% for BapA from PA7 strain as compared to PAO1 strain.
(TIF)

Figure S5 PAO1_GFP_ infected *Drosophila* guts. Flies were orally infected with GFP expressing PAO1 strain (green); guts were dissected at one day post-infection and stained with Alexa 546-phalloidin (actin, red) and DAPI (DNA, blue). Confocal sections (**A**) of anterior part of the gut showed bacterial high density area in the crop and proventriculus (PV). Magnifications (**B, C**) of indicated square showed high density bacteria sticking along the *Drosophila* epithelium.
(TIF)

Figure S6 Confocal sections of *Drosophila* infected proventriculus. Flies were orally infected with GFP expressing bacteria (green); guts were dissected at one day post-infection and stained with Alexa 546-phalloidin (actin, red) and DAPI (DNA, blue). PAO1_GFP_ (**A**, **B**), PprBK_GFP_ (**C**, **D**) and PprBKΔ*bapA*_GFP_ (**E**, **F**). Entire views of the proventriculus (PV) (**A**, **C**, **E**); magnifications of indicated squares show bacteria accumulating in the lumen (**B**, **C**, **F**). Arrow shows dense bacterial aggregates which are likely to correspond to *in vivo* biofilms in the case of PprBK (**C**).
(TIF)

Figure S7 The PprB-6His version used for EMSA has been checked to be able to complement a *pprB* mutant for PprB-dependent expression of the reporter fusions *cupE–lacZ* (**A**) and *flp–lacZ* (**B**) and PprB-dependent CupE1 (**A**) and Flp (**B**) production. Number on left side is molecular weight standard (kDa).
(TIF)

Table S1 Genes differentially expressed in Experiment 1 comparing PAO1*attB::cupE-lacZ*/pMMB*pprB* strain vs PAO1*attB::cupE-lacZ*/pMMB67HE strain.
(DOC)

Table S2 Genes differentially expressed in Experiment 2 comparing PAO1*attB::cupE-lacZ* strain vs PAO1Δ*pprB attB::cupE-lacZ* strain.
(DOC)

Table S3 Antibiotic susceptibility of *P. aeruginosa* strains. ^a^ The data presented have been obtained on five distinct and independent experiments and MIC, MBC-P (minimal bactericidal concentrations for planktonic cells) or MBC-B (minimal bactericidal concentrations for biofilm cells) values were found to be strictly identical in the five different experiments.
(DOC)

Table S4 Strains used in this study. ^*^Sm^R^, streptomycin resistance, Ap^R^, ampicillin resistance, Km^R^, kanamycin resistance, Gm^R^ gentamicin resistance, Tc^R^ tetracycline resistance.
(DOC)

Table S5 Plasmids used in this study. ^*^Sm^R^, streptomycin resistance; Ap^R^, ampicillin resistance; Km^R^, kanamycin resistance; Gm^R^ gentamicin resistance; Tc^R^ tetracycline resistance.
(DOC)

Table S6 Oligonucleotides used for mutagenesis and gene cloning.
(DOC)

Text S1 Supplemental experimental procedures.
(DOC)
