# Correction: Unique Biofilm Signature, Drug Susceptibility and Decreased Virulence in *Drosophila* through the *Pseudomonas aeruginosa* Two-Component System PprAB

**DOI:** 10.1371/annotation/5c169544-7d19-40db-9a58-28b2fdf2c82c

**Published:** 2012-12-12

**Authors:** Sophie de Bentzmann, Caroline Giraud, Christophe S. Bernard, Virginie Calderon, Friederike Ewald, Patrick Plésiat, Cathy Nguyen, Didier Grunwald, Ina Attree, Katy Jeannot, Marie-Odile Fauvarque, Christophe Bordi

An error was introduced in the preparation of this article for publication. The subheading "A new large cell surface and secreted adhesion" should instead read "A new large cell surface and secreted adhesin".

