# Supplementary figures and images for: Unique Biofilm Signature, Drug Susceptibility and Decreased Virulence in Drosophila through the Pseudomonas aeruginosa Two-Component System PprAB
Source: PLoS Pathog. 2012 Nov 29;8(11):e1003052. doi: 10.1371/journal.ppat.1003052 (PMC3510237; doi:10.1371/journal.ppat.1003052)

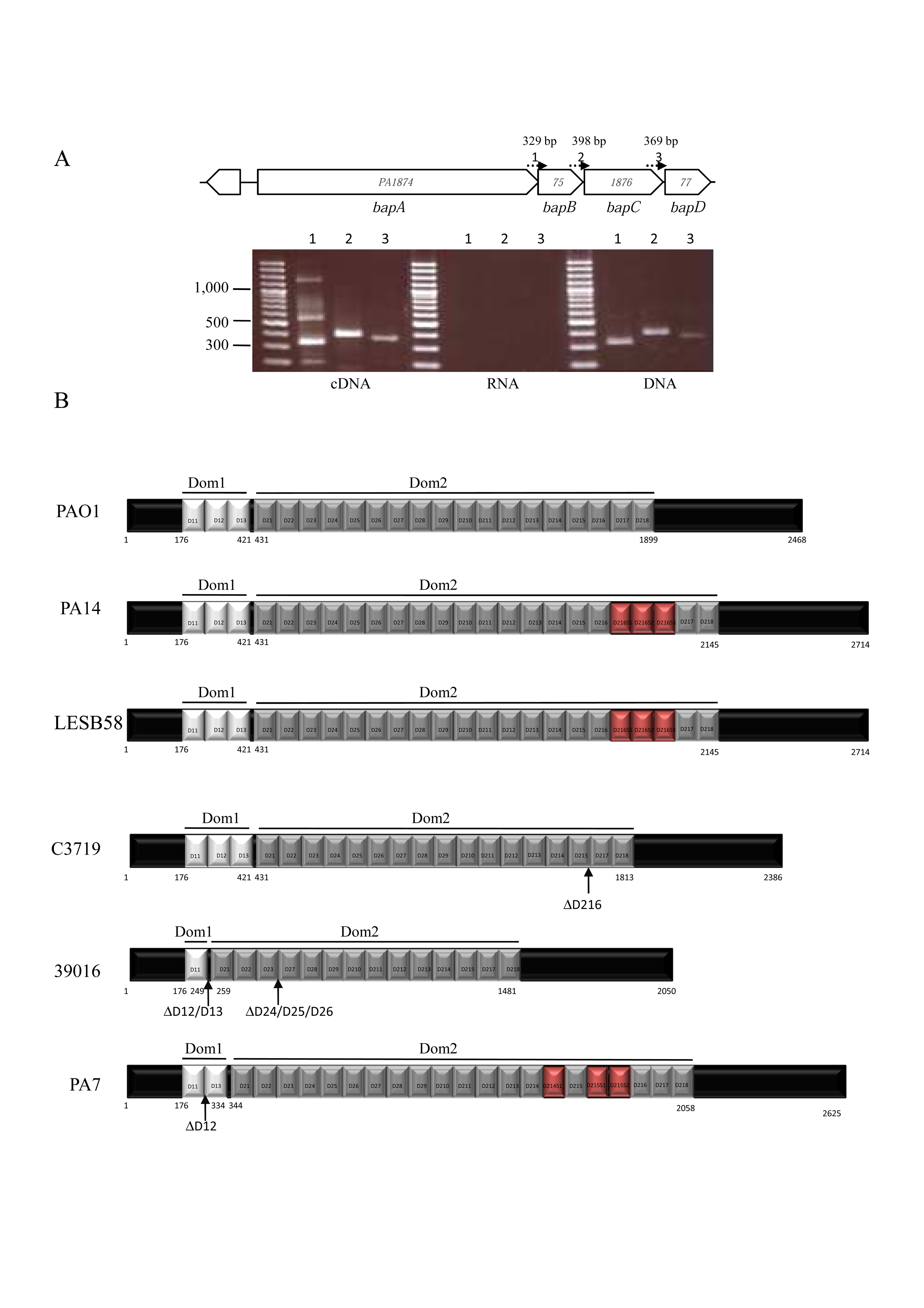

Supplement: Figure S4 — Genetic organization (A, Upper panel) of the P. aeruginosa bapABCD locus. Above is the precise localization of primers used to target each contiguous gene junction (see Table S6) for determination of operonic structure along the locus. Corresponding bands (A, Lower panel) with expected size obtained after PCR on genomic DNA were compared to the ones obtained after RT-PCR on RNA (cDNA). Absence of DNA contamination of RNA was controlled. DNA ladder and sizes in bp are precised on the left. Analysis of BapA sequences (B) from PA14, LESB58, C3719, 39016 and PA7 strains performed using Xstream and compared to the BapA sequence from PAO1 strain. Deletions were observed in Domain 1 for 39016 (D12/D13) and PA7 (D12) strains. In Domain 2, insertions of repeats D216S1/D216S2/D216S3 in PA14 and LESB58 strains, D214S1 and D215S1/D215S2 in PA7 strain and deletions of D216 for C3719 strain and D24/D25/D26 for 39016 strain were observed. Polymorphism raises 22% for BapA from PA7 strain as compared to PAO1 strain. (TIF) [file ppat.1003052.s004.tif]

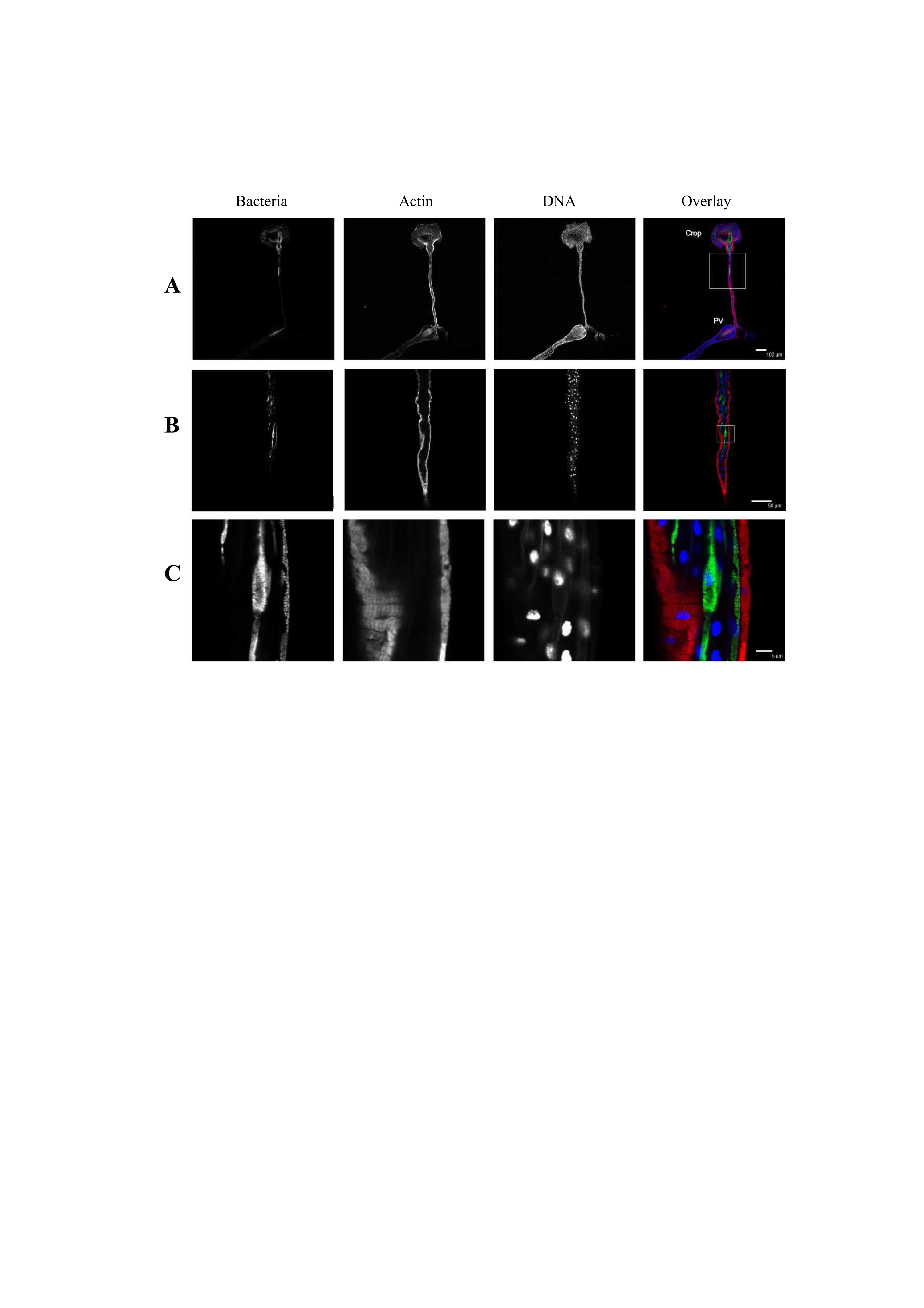

Supplement: Figure S5 — PAO1GFP infected Drosophila guts. Flies were orally infected with GFP expressing PAO1 strain (green); guts were dissected at one day post-infection and stained with Alexa 546-phalloidin (actin, red) and DAPI (DNA, blue). Confocal sections (A) of anterior part of the gut showed bacterial high density area in the crop and proventriculus (PV). Magnifications (B, C) of indicated square showed high density bacteria sticking along the Drosophila epithelium. (TIF) [file ppat.1003052.s005.tif]

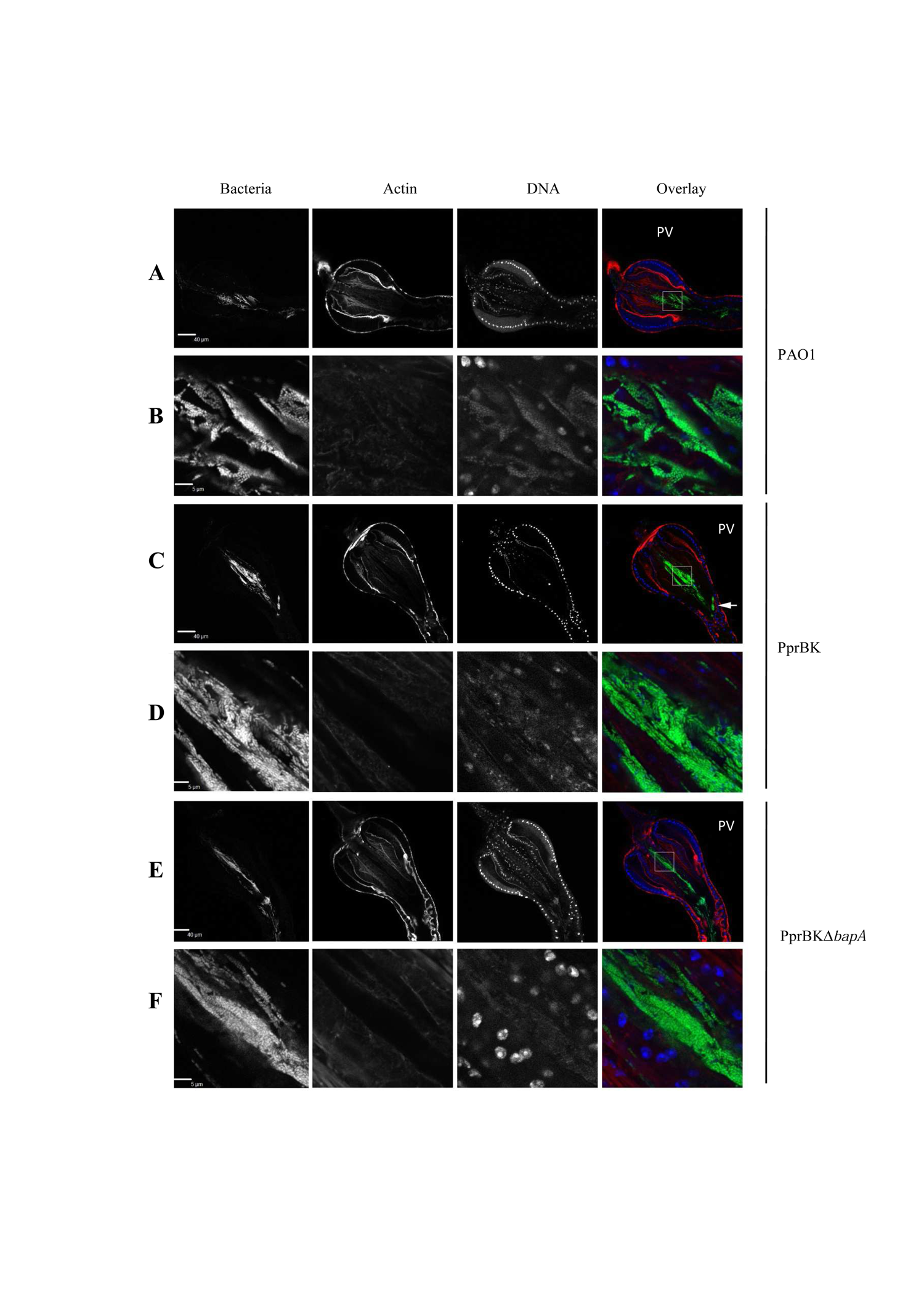

Supplement: Figure S6 — Confocal sections of Drosophila infected proventriculus. Flies were orally infected with GFP expressing bacteria (green); guts were dissected at one day post-infection and stained with Alexa 546-phalloidin (actin, red) and DAPI (DNA, blue). PAO1GFP (A, B), PprBKGFP (C, D) and PprBKΔbapA GFP (E, F). Entire views of the proventriculus (PV) (A, C, E); magnifications of indicated squares show bacteria accumulating in the lumen (B, C, F). Arrow shows dense bacterial aggregates which are likely to correspond to in vivo biofilms in the case of PprBK (C). (TIF) [file ppat.1003052.s006.tif]
