# Supplementary material for: Unique Biofilm Signature, Drug Susceptibility and Decreased Virulence in Drosophila through the Pseudomonas aeruginosa Two-Component System PprAB
Source: PLoS Pathog. 2012 Nov 29;8(11):e1003052. doi: 10.1371/journal.ppat.1003052 (PMC3510237; doi:10.1371/journal.ppat.1003052)
Supplement: Table S1 — Genes differentially expressed in Experiment 1 comparing PAO1attB::cupE-lacZ/pMMBpprB strain vs PAO1attB::cupE-lacZ/pMMB67HE strain. (DOC) [file ppat.1003052.s008.doc]

| Table S1. Genes differentially expressed in Experiment 1 comparing PAO1*attB::cupE-lacZ*/pMMB*pprB* strain vs PAO1*attB::cupE-lacZ*/pMMB67HE strain | | | | |
| --- | --- | --- | --- | --- |
| PA number | fold induction | | function |  |
| PA0040 | - | 4,59 | hypothetical protein |  |
| PA0041 | - | 3,95 | probable hemagglutinin |  |
| PA0603 | - | 2,09 | probable ATP-binding component of ABC transporter |  |
| PA0604 | - | 2,15 | probable binding protein component of ABC transporter |  |
| PA0779 | + | 2,35 | probable ATP-dependent protease |  |
| PA0852 | + | 2,15 | chitin-binding protein CbpD precursor |  |
| PA0878 | + | 2,28 | hypothetical protein |  |
| PA0879 | + | 2,28 | probable acyl-CoA dehydrogenase |  |
| PA0880 | + | 2,31 | probable ring-cleaving dioxygenase |  |
| PA0882 | + | 2,17 | hypothetical protein |  |
| PA0883 | + | 2,15 | probable acyl-CoA lyase beta chain |  |
| PA0996 | + | 3,28 | probable coenzyme A ligase PqsA |  |
| PA0997 | + | 3,28 | PqsB |  |
| PA0998 | + | 3,45 | PqsC |  |
| PA0999 | + | 4,02 | 3-oxoacyl-[acyl-carrier-protein] synthase III or PqsD |  |
| PA1000 | + | 3,66 | Quinolone signal response protein or PqsE |  |
| PA1001 | + | 3,74 | anthranilate synthase component I or PhnA |  |
| PA1002 | + | 3,59 | anthranilate synthase component II or PhnB |  |
| PA1194 | - | 2,26 | probable amino acid permease |  |
| PA1195 | - | 3,67 | hypothetical protein |  |
| PA1213 | + | 2 | putative clavaminic acid synthetase |  |
| PA1214 | + | 2,16 | putative sparagine synthase |  |
| PA1216 | + | 2,39 | hypothetical protein |  |
| PA1217 | + | 2,39 | probable 2-isopropylmalate synthase |  |
| PA1218 | + | 2,21 | hypothetical protein |  |
| PA1416 | - | 2,08 | hypothetical protein |  |
| PA1417 | - | 2,88 | probable decarboxylase |  |
| PA1418 | - | 4,05 | probable sodium:solute symport protein |  |
| PA1419 | - | 6,24 | probable transporter |  |
| PA1420 | - | 8,09 | hypothetical protein |  |
| PA1421 | - | 5,41 | guanidinobutyrase |  |
| PA1485 | + | 2,29 | probable amino acid permease |  |
| PA1632 | - | 2,09 | KdpF |  |
| PA1804 | + | 2,05 | DNA-binding protein HU |  |
| PA1871 | + | 2,15 | LasA protease precursor |  |
| PA1874 | + | 25,72 | BapA |  |
| PA1875 | + | 28,81 | BapB |  |
| PA1876 | + | 19,53 | BapC |  |
| PA1877 | + | 16,94 | BapD |  |
| PA1878 | - | 2,01 | hypothetical protein |  |
| PA1914 | + | 31,86 | HvnA |  |
| PA1979 | + | 2,22 | sensor kinase, EraS |  |
| PA2018 | - | 3,25 | RND multidrug efflux transporter |  |
| PA2019 | - | 2,78 | RND multidrug efflux membrane fusion protein precursor |  |
| PA2062 | + | 2,23 | probable pyridoxal-phosphate dependent enzyme |  |
| PA2066 | + | 2,3 | hypothetical protein |  |
| PA2067 | + | 2,23 | probable hydrolase |  |
| PA2069 | + | 2,45 | probable carbamoyl transferase |  |
| PA2073 | - | 3,42 | probable transporter (membrane subunit) |  |
| PA2074 | - | 3,86 | hypothetical protein |  |
| PA2083 | + | 4,71 | probable ring-hydroxylating dioxygenase subunit |  |
| PA2134 | - | 2,22 | hypothetical protein |  |
| PA2146 | - | 3,27 | hypothetical protein |  |
| PA2160 | - | 2,02 | probable glycosyl hydrolase |  |
| PA2161 | - | 2,25 | hypothetical protein |  |
| PA2162 | - | 2,08 | probable glycosyl hydrolase |  |
| PA2166 | - | 2,04 | hypothetical protein |  |
| PA2184 | - | 2,04 | hypothetical protein |  |
| PA2205 | + | 3,47 | hypothetical protein |  |
| PA2222 | + | 2,26 | hypothetical protein |  |
| PA2223 | + | 2,05 | hypothetical protein |  |
| PA2300 | + | 3,16 | chitinase or ChiC |  |
| PA2570 | + | 2,27 | PA-I galactophilic lectin or LecA |  |
| PA2593 | - | 2,09 | quorum threshold expression element, QteE |  |
| PA2792 | + | 2,66 | hypothetical protein |  |
| PA2793 | + | 3 | hypothetical protein |  |
| PA3049 | - | 10,32 | ribosome modulation factor rmF |  |
| PA3327 | - | 3,74 | probable non-ribosomal peptide synthetase |  |
| PA3328 | - | 3,98 | probable FAD-dependent monooxygenase |  |
| PA3329 | - | 3,44 | hypothetical protein |  |
| PA3330 | - | 3,09 | probable short chain dehydrogenase |  |
| PA3331 | - | 2,84 | cytochrome P450 |  |
| PA3332 | - | 2,85 | hypothetical protein |  |
| PA3333 | - | 2,74 | 3-oxoacyl-[acyl-carrier-protein] synthase III or FabH2 |  |
| PA3334 | - | 2,36 | probable acyl carrier protein |  |
| PA3335 | - | 2,37 | hypothetical protein |  |
| PA3336 | - | 2,34 | probable MFS transporter |  |
| PA3361 | + | 2,82 | PA-II fucose lectin or LecB |  |
| PA3368 | + | 2,29 | probable acetyltransferase |  |
| PA3520 | + | 2 | hypothetical protein |  |
| PA3641 | - | 2,08 | probable amino acid permease |  |
| PA3662 | - | 9,5 | hypothetical protein |  |
| PA3734 | + | 2,61 | hypothetical protein |  |
| PA3862 | - | 2,88 | DauB |  |
| PA3863 | - | 2,95 | DauA |  |
| PA3864 | - | 2,22 | DauR |  |
| PA3865 | - | 4,57 | probable amino acid binding protein |  |
| PA4034 | + | 3,11 | aquaporin Z or AqpZ |  |
| PA4141 | - | 3,51 | hypothetical protein |  |
| PA4142 | - | 2,51 | probable secretion protein |  |
| PA4147 | - | 2,01 | transcriptional regulator AcoR |  |
| PA4171 | - | 2,15 | probable protease |  |
| PA4175 | + | 6,84 | Pvds-regulated endoprotease, lysyl class or PrpL |  |
| PA4217 | + | 2,36 | flavin-containing monooxygenase or PhzS |  |
| PA4293 | + | 11,5 | two-component sensor PprA |  |
| PA4294 | + | 15,68 | TadF |  |
| PA4295 | + | 2,27 | FppA |  |
| PA4296 | + | 32,23 | two-component response regulator PprB |  |
| PA4297 | + | 3,86 | TadG |  |
| PA4298 | + | 4,35 | hypothetical protein |  |
| PA4299 | + | 5,13 | TadD |  |
| PA4300 | + | 5,51 | TadC |  |
| PA4301 | + | 4,46 | TadB |  |
| PA4302 | + | 5,49 | TadA |  |
| PA4303 | + | 4,36 | TadZ |  |
| PA4304 | + | 5,33 | RcpA |  |
| PA4305 | + | 5,33 | RcpC |  |
| PA4306 | + | 5,6 | Flp |  |
| PA4507 | - | 2,1 | hypothetical protein |  |
| PA4563 | - | 2,13 | 30S ribosomal protein S20 |  |
| PA4648 | - | 5,67 | CupE1 |  |
| PA4649 | + | 7,98 | CupE2 |  |
| PA4650 | + | 6,75 | CupE3 |  |
| PA4651 | + | 7,74 | CupE4 |  |
| PA4652 | + | 2,12 | CupE5 |  |
| PA4676 | + | 2,13 | probable carbonic anhydrase |  |
| PA4677 | + | 2,43 | 30S ribosomal protein S6 or RpsF |  |
| PA4986 | - | 2,03 | probable oxidoreductase |  |
| PA5096 | - | 2,68 | probable binding protein component of ABC transporter |  |
| PA5097 | + | 4,3 | probable amino acid permease |  |
| PA5098 | + | 4,97 | histidine ammonia-lyase or HutH |  |
| PA5099 | + | 4,13 | probable transporter |  |
| PA5100 | + | 7,22 | urocanase or HutU |  |
| PA5104 | + | 2,83 | hypothetical protein |  |
| PA5105 | + | 2,75 | histidine utilization repressor HutC |  |
| PA5106 | + | 5,53 | HutF |  |
| PA5287 | + | 2,28 | ammonium transporter AmtB |  |
| PA5368 | - | 2,15 | membrane protein component of ABC phosphate transporter or PstC |  |
| PA5369 | - | 2,44 | ABC transporter, periplasmic phosphate-binding protein, PstS |  |
| PA5429 | + | 2,19 | aspartate ammonia-lyase or AspA |  |
