# Supplementary material for: Unique Biofilm Signature, Drug Susceptibility and Decreased Virulence in Drosophila through the Pseudomonas aeruginosa Two-Component System PprAB
Source: PLoS Pathog. 2012 Nov 29;8(11):e1003052. doi: 10.1371/journal.ppat.1003052 (PMC3510237; doi:10.1371/journal.ppat.1003052)
Supplement: Table S2 — Genes differentially expressed in Experiment 2 comparing PAO1attB::cupE-lacZ strain vs PAO1ΔpprB attB::cupE-lacZ strain. (DOC) [file ppat.1003052.s009.doc]

| Table S2. Genes differentially expressed in Experiment 2 comparing PAO1*attB::cupE-lacZ* strain vs PAO1Δ*pprB attB::cupE-lacZ* strain | | | | | | | |
| --- | --- | --- | --- | --- | --- | --- | --- |
| PA number | fold induction | | function |  |  |  |  |
| PA0866 | - | 4,75 | aromatic amino acid transport protein AroP2 |  |  |  |  |
| PA0998 | - | 3,27 | PqsC |  |  |  |  |
| PA0999 | - | 3,6 | 3-oxoacyl-[acyl-carrier-protein] synthase III or PqsD |  |  |  |  |
| PA1000 | - | 3,43 | Quinolone signal response protein or PqsE |  |  |  |  |
| PA1001 | - | 3,45 | anthranilate synthase component I or PhnA |  |  |  |  |
| PA1002 | - | 3,4 | anthranilate synthase component II or PhnB |  |  |  |  |
| PA1155 | + | 2,099 | NrdB, tyrosyl radical-harboring component of class Ia ribonucleotide reductase |  |  |  |  |
| PA1213 | - | 2,2 | putative clavaminic acid synthetase |  |  |  |  |
| PA1214 | - | 2,59 | putative sparagine synthase |  |  |  |  |
| PA1215 | - | 2,39 | hypothetical protein |  |  |  |  |
| PA1216 | - | 2,15 | hypothetical protein |  |  |  |  |
| PA1217 | - | 2,532 | probable 2-isopropylmalate synthase |  |  |  |  |
| PA1218 | - | 2,6 | hypothetical protein |  |  |  |  |
| PA1219 | - | 2,21 | hypothetical protein |  |  |  |  |
| PA1220 | - | 2,39 | hypothetical protein |  |  |  |  |
| PA1221 | - | 2,49 | hypothetical protein |  |  |  |  |
| PA1705 | + | 2,42 | regulator in type III secretion |  |  |  |  |
| PA1706 | + | 2 | type III secretion protein PcrV |  |  |  |  |
| PA1707 | + | 2,55 | regulatory protein PcrH |  |  |  |  |
| PA1708 | + | 2,26 | translocator protein PopB |  |  |  |  |
| PA1709 | + | 2,26 | Translocator outer membrane protein PopD precursor |  |  |  |  |
| PA1710 | + | 2,6 | ExsC, exoenzyme S synthesis protein C precursor |  |  |  |  |
| PA1711 | + | 2,13 | ExsE |  |  |  |  |
| PA1714 | + | 2,12 | ExsD |  |  |  |  |
| PA1874 | - | 12,64 | BapA |  |  |  |  |
| PA1875 | - | 9,17 | BapB |  |  |  |  |
| PA1876 | - | 6,41 | BapC |  |  |  |  |
| PA1877 | - | 7,11 | BapD |  |  |  |  |
| PA1914 | - | 3,87 | HvnA |  |  |  |  |
| PA2432 | - | 2,47 | bistable expression regulator, BexR |  |  |  |  |
| PA2513 | + | 10,66 | anthranilate dioxygenase small subunit |  |  |  |  |
| PA2697 | - | 2,23 | hypothetical protein |  |  |  |  |
| PA2698 | - | 3,01 | probable hydrolase |  |  |  |  |
| PA3221 | - | 2,91 | CsaA |  |  |  |  |
| PA3222 | - | 3,4 | hypothetical protein |  |  |  |  |
| PA3662 | + | 3,73 | hypothetical protein |  |  |  |  |
| PA4293 | - | 4,45 | two-component sensor PprA |  |  |  |  |
| PA4294 | - | 4,04 | TadF |  |  |  |  |
| PA4296 | - | 38,5 | two-component response regulator PprB |  |  |  |  |
| PA4297 | - | 20,24 | TadG |  |  |  |  |
| PA4298 | - | 10,76 | hypothetical protein |  |  |  |  |
| PA4299 | - | 16,84 | TadD |  |  |  |  |
| PA4300 | - | 15,8 | TadC |  |  |  |  |
| PA4301 | - | 11,05 | TadB |  |  |  |  |
| PA4302 | - | 19,17 | TadA |  |  |  |  |
| PA4303 | - | 9,95 | TadZ |  |  |  |  |
| PA4304 | - | 13,45 | RcpA |  |  |  |  |
| PA4305 | - | 17 | RcpC |  |  |  |  |
| PA4306 | - | 39 | Flp |  |  |  |  |
| PA4648 | - | 7,03 | CupE1 |  |  |  |  |
| PA4649 | - | 11,85 | CupE2 |  |  |  |  |
| PA4650 | - | 7,45 | CupE3 |  |  |  |  |
| PA4651 | - | 11,7 | CupE4 |  |  |  |  |
| PA4652 | - | 3,53 | CupE5 |  |  |  |  |
| PA4653 | - | 3,17 | CupE6 |  |  |  |  |
| PA5287 | - | 2,21 | ammonium transporter AmtB |  |  |  |  |
