# Supplementary material for: Unique Biofilm Signature, Drug Susceptibility and Decreased Virulence in Drosophila through the Pseudomonas aeruginosa Two-Component System PprAB
Source: PLoS Pathog. 2012 Nov 29;8(11):e1003052. doi: 10.1371/journal.ppat.1003052 (PMC3510237; doi:10.1371/journal.ppat.1003052)
Supplement: Table S3 — Antibiotic susceptibility of P. aeruginosa strains. a The data presented have been obtained on five distinct and independent experiments and MIC, MBC-P (minimal bactericidal concentrations for planktonic cells) or MBC-B (minimal bactericidal concentrations for biofilm cells) values were found to be strictly identical in the five different experiments. (DOC) [file ppat.1003052.s010.doc]

*Table S3. Antibiotic susceptibility of P. aeruginosa strains*.

|  | tobramycin | | |  | ciprofloxacin | |
| --- | --- | --- | --- | --- | --- | --- |
|  | MIC^a^  (μg/ml) | MBC-P^a^  (μg/ml) | MBC-B^a^  (μg/ml) | MIC^a^  (μg/ml) | MBC-P^a^  (μg/ml) | MBC-B^a^  (μg/ml) |
| PAO1 | 0.5 | 64 | 512 | 0.12 | 4 | 64 |
| PAO1Δ*pprB* | 0.5 | 64 | 512 | 0.12 | 2 | 64 |
| PprBK | 0.25 | 32 | 256 | 0.12 | 2 | 64 |
| PAO1Δ*bapD* | 0.5 | 64 | 512 | 0.12 | 8 | 64 |
| PprBKΔ*bapD* | 0.25 | 32 | 256 | 0.12 | 8 | 128 |

^a^ The data presented have been obtained on five distinct and independent experiments and MIC, MBC-P (minimal bactericidal concentrations for planktonic cells) or MBC-B (minimal bactericidal concentrations for biofilm cells) values were found to be strictly identical in the five different experiments.
