# Supplementary material for: Unique Biofilm Signature, Drug Susceptibility and Decreased Virulence in Drosophila through the Pseudomonas aeruginosa Two-Component System PprAB
Source: PLoS Pathog. 2012 Nov 29;8(11):e1003052. doi: 10.1371/journal.ppat.1003052 (PMC3510237; doi:10.1371/journal.ppat.1003052)
Supplement: Table S4 — Strains used in this study. *SmR, streptomycin resistance, ApR, ampicillin resistance, KmR, kanamycin resistance, GmR gentamicin resistance, TcR tetracycline resistance. (DOC) [file ppat.1003052.s011.doc]

Table S4. Strains used in this study

| Strain | Relevant characteristics^*^ | Source |
| --- | --- | --- |
| *E. coli*  TG1  TOP10F’  CC118(λpir)  pRK2013  *P. aeruginosa*  PAO1  PAO1Δ*hvnA*  PAO1Δ*pel*  PAO1Δ*psl*  PprBK  PprBKΔ*bapA*  PprBKΔ*bapD*  PprBKΔ*cupE5*  PprBKΔ*flp*  PprBKΔ*cupE5*Δ*bapA*Δ*flp*  PprBKΔ*pel*  PprBKΔ*psl*  PAO1_GFP_  PprBK _GFP_  PprBKΔ*bapA* _GFP_  PprBKΔ*bapD* _GFP_  PprBKΔ*cupE5* _GFP_  PprBKΔ*flp* _GFP_  PprBKΔ*cupE5*Δ*bapA*Δ*flp*_GFP_  PAO1Δ*pprB*  PAO1*attB*::*cupE-lacZ*  PAO1Δ*pprB* *attB*::*cupE-lacZ*  PAO1*attB*::*rcpC-lacZ*  PAO1Δ*pprB* *attB*::*rcpC-lacZ*  PAO1*attB*::*bapA -lacZ*  PAO1Δ*pprB* *attB*::*bapA -lacZ*  PAO1*attB*::*hvnA -lacZ*  PAO1Δ*pprB* *attB*::*hvnA -lacZ*  PAO1*attB*::*pqsA -lacZ*  PAO1Δ*pprB* *attB*::*pqsA -lacZ*  PAO1*attB*::*phnA -lacZ*  PAO1*attB*::*PA1215 –lacZ*  PAO1*attB*::*PA1221 –lacZ*  PAO1*attB*::*PA3662 -lacZ*  PAO1Δ*pprB* *attB*::*PA3662 -lacZ*  PAO1*attB*::*glnK -lacZ* | *supE* Δ(*lac-proAB*) *thi* *hsdR*Δ*5* (F’ *traD36* *rpoA*^+^ *B^+^ lacI*^q^ *ZΔM15*)  F’ (*lacI*^q^ Tn10 (Tet^R^)) *mrcA Δ(mrr-hsdRMS-mcrBC*) Φ80 *lacZ*Δ*M15* Δ*lacX74 recA1*  Host strain for pKNG101 replication, Δ(*ara-leu*) *araD* Δl*ac*X74 *galE* *galK phoA20 thi-1 rpsE rpoB* *argE*(Am) *recA1* Rf^R^ (λpir)  ColE1 *ori*, *tra^+^*, *mob^+^*, Km^R^  Wild type (WT)  PAO1 deletion mutant for the *hvnA* gene  PAO1 deletion mutant for the *pelC* gene  PAO1 deletion mutant for the *pslCD* genes  PAO1_LB_Tn10 mutant with the p*tac* promoter located upstream from the *pprB* gene, Gm^R^, Tc^R^  PprBK deletion mutant for the *bapA* gene, Gm^R^, Tc^R^  PprBK deletion mutant for the *bapD* gene, Gm^R^, Tc^R^  PprBK deletion mutant for the *cupE5* gene, Gm^R^, Tc^R^  PprBK deletion mutant for the *flp* gene, Gm^R^, Tc^R^  PprBK deletion mutant for the *cupE5, bapA and flp* genes, Gm^R^, Tc^R^  PprBK deletion mutant for the *pelC* gene, Gm^R^, Tc^R^  PprBK deletion mutant for the *pslCD* genes, Gm^R^, Tc^R^  PAO1 strain tagged with EGFP in a mini Tn7 construct ; Sm^R^  PprBK strain tagged with EGFP in a mini Tn7 construct ; Gm^R^, Tc^R^, Sm^R^  PprBKΔ*bapA* strain tagged with EGFP in a mini Tn7 construct ; Gm^R^, Tc^R^, Sm^R^  PprBKΔ*bapD*strain tagged with EGFP in a mini Tn7 construct; Gm^R^, Tc^R^, Sm^R^ PprBKΔ*cupE5* strain tagged with EGFP in a mini Tn7 construct; Gm^R^, Tc^R^, Sm^R^  PprBKΔ*flp* strain tagged with EGFP in a mini Tn7 construct ; Gm^R^, Tc^R^, Sm^R^  PprBKΔ*cupE5*Δ*bapA*Δ*flp* strain tagged with EGFP in a mini Tn7 construct ; Gm^R^, Tc^R^, Sm^R^  PAO1 deletion mutant for the *pprB* gene  PAO1 strain with *cupE-lacZ* fusion inserted at *attB* sites, Tc^R^  PAO1Δ*pprB* strain with *cupE-lacZ* fusion inserted at *attB* sites, Tc^R^  PAO1 strain with *rcpC-lacZ* fusion inserted at *attB* sites, Tc^R^  PAO1Δ*pprB* strain with *rcpC-lacZ* fusion inserted at *attB* sites, Tc^R^  PAO1 strain with *PA1874-lacZ* fusion inserted at *attB* sites, Tc^R^  PAO1Δ*pprB* strain with *bapA-lacZ* fusion inserted at *attB* sites, Tc^R^  PAO1 strain with *hvnA-lacZ* fusion inserted at *attB* sites, Tc^R^  PAO1Δ*pprB* strain with *hvnA-lacZ* fusion inserted at *attB* sites, Tc^R^  PAO1 strain with *pqsA-lacZ* fusion inserted at *attB* sites, Tc^R^  PAO1Δ*pprB* strain with *pqsA-lacZ* fusion inserted at *attB* sites, Tc^R^  PAO1 strain with *phnA-lacZ* fusion inserted at *attB* sites, Tc^R^  PAO1 strain with *PA1215-lacZ* fusion inserted at *attB* sites, Tc^R^  PAO1 strain with *PA1221-lacZ* fusion inserted at *attB* sites, Tc^R^  PAO1 strain with *PA3662-lacZ* fusion inserted at *attB* sites, Tc^R^  PAO1Δ*pprB* strain with *PA3662-lacZ* fusion inserted at *attB* sites, Tc^R^  PAO1 strain with *glnK-lacZ* fusion inserted at *attB* sites, Tc^R^ | Lab collection  Invitrogen  Lab collection  Lab collection  Lab collection  This study  This study  This study  This study  This study  This study  This study  This study  This study  This study  This study  This study  This study  This study  This study  This study  This study  This study  This study  This study  This study  This study  This study  This study  This study  This study  This study  This study  This study |

^*^Sm^R^, streptomycin resistance, Ap^R^, ampicillin resistance, Km^R^, kanamycin resistance, Gm^R^ gentamicin resistance, Tc^R^ tetracycline resistance
