# Supplementary material for: Unique Biofilm Signature, Drug Susceptibility and Decreased Virulence in Drosophila through the Pseudomonas aeruginosa Two-Component System PprAB
Source: PLoS Pathog. 2012 Nov 29;8(11):e1003052. doi: 10.1371/journal.ppat.1003052 (PMC3510237; doi:10.1371/journal.ppat.1003052)
Supplement: Table S5 — Plasmids used in this study. *SmR, streptomycin resistance; ApR, ampicillin resistance; KmR, kanamycin resistance; GmR gentamicin resistance; TcR tetracycline resistance. (DOC) [file ppat.1003052.s012.doc]

Table S5. Plasmids used in this study

| Plasmid | Relevant characteristics^*^ | | Source |
| --- | --- | --- | --- |
| pCR2.1  pMMB67-HE  pKNG101  pMMB*pprB*  pMMB*pprB*6His  pYW024  miniTn7*gfp*  pRK600  pUX-BF13  miniCTX-*pcupE*-*lacZ*  miniCTX-*prcpC-lacZ*  miniCTX-*pbapA*-*lacZ*  miniCTX-*phvnA*-*lacZ*  miniCTX-*ppqsA*-*lacZ*  miniCTX-*pphnA*-*lacZ*  miniCTX-*pPA1215*-*lacZ*  miniCTX-*pPA1221*-*lacZ*  miniCTX-*pPA3662*-*lacZ*  miniCTX-*pglnK*-*lacZ*  pKNGDel*bapA*  pKNGDel*bapD*  pKNGDel*cupE5*  pKNGDel*flp*  pKNGDel*pprB*  pKNGDel*hvnA*  pKNGDel*pelC*  pKNGDel*pslCD* | TA cloning vector for PCR products, *lacZ*α ColE1 f1 *ori*, Ap^R^, Km^R^  Broad host range vector, IncQ; p*tac*; *lacZ*α, Ap^R^  Suicide vector in *P. aeruginosa*, *sacB^+^*, Sm^R^  *pprB* gene cloned in pMMB67-HE, Ap^R^  *pprB* gene with a His-tag cloned in pMMB67-HE at *Sac*I/*Xba*I, Ap^R^  *pprB* gene cloned in pQE31 vector with a His-tag, Ap^R^  *mob^+^ ori*; Ap^R^Sm^R^; miniTn7 vector containing *gfp*mut3 inserted into *notI* site  Cm^R^; ColE1 oriV, RP4 tra +, RP4 oriT  *mob^+^ ori-*R6K; Ap^R^; helper plasmid providing the Tn7 transposition functions in *trans*  Promoter region of *cupE* gene inserted into miniCTX-*lacZ*, Tc^R^  Promoter region of *rcpC* gene inserted into miniCTX-*lacZ*, Tc^R^  Promoter region of *bapA* gene inserted into miniCTX-*lacZ* at *Hin*dIII/*Bam*HI sites, Tc^R^  Promoter region of *hvnA* gene inserted into miniCTX-*lacZ* at *Hin*dIII/*Bam*HI sites, Tc^R^  Promoter region of *pqsA* gene inserted into miniCTX-*lacZ* at *Eco*RI/*Hin*dIII sites, Tc^R^  Promoter region of *phnA* gene inserted into miniCTX-*lacZ* at *Eco*RI/*Hin*dIII sites, Tc^R^  Promoter region of *PA1215* gene inserted into miniCTX-*lacZ* at *Eco*RI/*Hin*dIII sites, Tc^R^  Promoter region of *PA1221* gene inserted into miniCTX-*lacZ* at *Eco*RI/*Hin*dIII sites, Tc^R^  Promoter region of *PA3662* gene inserted into miniCTX-*lacZ* at *Bam*HI/*Eco*RI sites, Tc^R^  Promoter region of *glnK* gene inserted into miniCTX-*lacZ* at *Bam*HI/*Eco*RI sites, Tc^R^  *bapA* gene mutator cloned into pKNG101, Sm^R^; *sacB*^+^ at *Bam*HI/*Apa*I *sites*  *bapD*gene mutator cloned into pKNG101, Sm^R^; *sacB*^+^at *Bam*HI/*Apa*I *sites*  *cupE5* gene mutator cloned into pKNG101, Sm^R^; *sacB*^+^  *flp* gene mutator cloned into pKNG101, Sm^R^; *sacB*^+^  *pprB* gene mutator cloned into pKNG101, Sm^R^; *sacB*^+^  *hvnA* gene mutator cloned into pKNG101, Sm^R^; *sacB*^+^ at *Bam*HI/*Apa*I *sites*  *pelC* gene mutator cloned into pKNG101, Sm^R^; *sacB^+^*  *pslCD* gene mutator cloned into pKNG101 Sm^R^; *sacB^+^* at *Bam*HI/*Apa*I *sites* | Invitrogen  Lab collection  Lab collection  This study  Lab collection    This study  This study  This study  This study  This study  This study  This study  This study  This study  This study  This study  This study  and this study | |

^*^Sm^R^, streptomycin resistance; Ap^R^, ampicillin resistance; Km^R^, kanamycin resistance; Gm^R^ gentamicin resistance; Tc^R^ tetracycline resistance.
