# Supplementary material for: Unique Biofilm Signature, Drug Susceptibility and Decreased Virulence in Drosophila through the Pseudomonas aeruginosa Two-Component System PprAB
Source: PLoS Pathog. 2012 Nov 29;8(11):e1003052. doi: 10.1371/journal.ppat.1003052 (PMC3510237; doi:10.1371/journal.ppat.1003052)
Supplement: Table S6 — Oligonucleotides used for mutagenesis and gene cloning. (DOC) [file ppat.1003052.s013.doc]

Table S6. Oligonucleotides used for mutagenesis and gene cloning

|  | | Oligonucleotide (5’→3’) |  |
| --- | --- | --- | --- |
|  | Mutations  *bapA*  DelBapAUp5  DelBapAUp3  DelBapADn5  DelBapADn3  *bapD*  DelBapDUp5  DelBapDUp3  DelBapDDn5  DelBapDDn3  *hvnA*  DelHvnAUp5  DelHvnAUp3  DelHvnADn5  DelHvnADn3  *psl*  DelPslUp  DelPslDn  *bap* operon  *bapA-bapB* junction  *bapB-bapC* junction  *bapC-bapD* junction | AGAGGATCGGTTGGATAACTTGG  CGCATCACATTTCCGACATTCCTCTTGTC  GGAAATGTGATGCGCGGGCGCAGG  GGGTCATCTCGCGGATGCC  CTGTTCCATGGCAGCCTGC  CGCCTCATCAGAGTCCATGGTTGCCTC  ACTCTGATGAGGCGTGCAGGACAGG  AAGCGATCCACGGCGACATC  TTGCGAATGCATTAATCCCCCTT  GGCCTCACATGGGATGGACTCCACGCA  TCCCATGTGAGGCCGCTCCGCGGGGG  AGTACCGTTTCCAGCAGCTTGC  GATCTCCATCACCGTCGAG  CAGCAAGCGCCTGGCCGAC  GCCAACAACACGTTGCAGATCAC  CGGCCATCGCGGATCGTACT  CTGTTGGGTCCGCTATTGGA  AGCGGCATCCTCTGGCTTC CGACTACCTGCAGCAATGGT AACGGCACGACCCGACC | |
| Chromosomal fusions  *bapA-lacZ*  PromBapAUp  PromBapADn  *hvnA-lacZ*  PromHvnAUp  PromHvnADn  *pqsA-lacZ*  PromPqsAUp  PromPqsADn  *phnA-lacZ*  PromPhnAUp  PromPhnADn  *PA1215-lacZ*  PromPA1215Up  PromPA1215Dn  *PA1221-lacZ*  PromPA1221Up  PromPA1221Dn  *PA3662-lacZ*  PromPA3662Up  PromPA3662Dn  *glnk-lacZ*  PromGlnKUp  PromGlnKDn  RT-qPCR | | CCCAAGCTTATACGCATCGTAGAGTTCTGA  CGGGATCCATAGGGGTAACTTTCGCCTG  CGGGATCCGCGGGTGTCAGCGCCAGGC  CCCAAGCTTGGCGGAGCCCGATACCTGG  CCCAAGCTTGCTCGCCCAGTGTACTACGC  GGAATTCTGATAAACGGCGGTATCGGG  CCCAAGCTTGATGCTGGAGATTCTCTCCC  GGAATTCTTCCAGTCGATAGCCAACCC  CCCAAGCTTGTGAACTTCGACATCCACGC  GGAATTCGATCAGCACCAGATGCTTGCC  CCAAGCTTCCGCCTCCTGAAAACCTCGG  GGAATTCGGACATTCTGACAATCGCC  GGAATTCGCACCACGACGATTCATCCC  CGGGATCCCTTCAGCAACAGCATCTCGG  CGGGGATCCACGTCGTCCAACTTGAACGG  GGAATTCGCAGTCTAGCACGATGTCGG |  |
| pqsA_RT_1  pqsA_RT_2  pelA_RT_1  pelA_RT_2  pslE_RT_1  pslE_RT_2  algD_RT_1  algD_RT_2  cupE1_RT_1  cupE1_RT_2  pprB_RT_1  pprB_RT_2  amtB_RT_1  amtB_RT_2  Up_UvrD  Do_UvrD | | ACCGTGATCAATCCCAAGTC  GCTGAACCAGGGAAAGAACA  ATCAAGCCCTATCCGTTCCT  AACGGATGGCTGAAGGTATG  GCGCACCAAGTTCTATGTGA  GCTCCAGGTGAAGCTGATCT  GGGCTATGTCGGTGCAGTAT  AACGATACGTCGGAGTCCAG  CGGCAATATCCAGATCCAGT  GAGATGTCCACCGGTGTGTT  TACGAGGTACACGGCAACAG  CCCAGCTCGTAGGCTATCTG  GGCTAGGAGCCCTATTGTCC  AGACCACCCAGAGAATGGTG  CACGCCTCGCCCTACAGCA  GGATCTGGAAGTTCTCGCTCAGC |  |
