# Supplementary material for: Unique Biofilm Signature, Drug Susceptibility and Decreased Virulence in Drosophila through the Pseudomonas aeruginosa Two-Component System PprAB
Source: PLoS Pathog. 2012 Nov 29;8(11):e1003052. doi: 10.1371/journal.ppat.1003052 (PMC3510237; doi:10.1371/journal.ppat.1003052)
Supplement: Text S1 — Supplemental experimental procedures. (DOC) [file ppat.1003052.s014.doc]

Supplemental experimental procedures

Bacterial strains and growth conditions. The strains and plasmids used in this study are listed in Tables S4 and S5, respectively. Strains were grown at 30°C or 37°C in M63 medium supplemented with 0.4% L-arginine and 1mM MgSO_4_ or in LB medium. When required, IPTG was added to a concentration of 10 µM. *E. coli* TG1 strain was used for standard genetic manipulations. Recombinant plasmids were introduced into *P. aeruginosa* through conjugative transfer, using pRK2013. Transconjugants were selected on *Pseudomonas* isolation agar (PIA) (Difco Laboratories) supplemented with appropriate antibiotics. The following antibiotic concentrations were used: 1) for *E. coli*: ampicillin (Ap), 50 µg/ml; gentamicin (Gm), 25 µg/ml; kanamycin (Km), 25 µg/ml; tetracycline (Tc), 15 µg/ml; streptomycin (Sm), 50 µg/ml; 2) for *P.* *aeruginosa*: carbenicillin (Cb), 300 µg/ml; gentamicin (Gm), 75 µg/ml; tetracycline (Tc), 200 µg/ml; streptomycin (Sm), 2 mg/ml.

Construction of *P. aeruginosa* deletion mutants. Fused upstream and downstream DNA regions of *bapA*, *bapD*, *hvnA* genes were PCR amplified in a two step procedure using appropriate oligonucleotide pairs, respectively (Table S6). The final DNA fragments of about 1.1 kb were inserted into the pCR2.1 vector. DNA fragments carrying appropriate restriction sites — *Bam*HI/*Apa*I —were further released by hydrolysis and inserted into the suicide vector pKNG101. The resulting recombinant plasmids as well as the ones obtained in previous studies containing mutators for *cupE5, flp* genes were then mobilized into *P. aeruginosa.* Additionally the *pel* mutator and a derived-*psl* mutator were also mobilized and the deletion mutants were selected on LB plates containing 5% sucrose and appropriate antibiotics as previously described . The initial *psl* mutator was reamplified by PCR using appropriate oligonucleotides (Table S6) cloned into pCR2.1 vector and further released by hydrolysis with *Bam*HI/*Apa*I and inserted into the suicide vector pKNG101.

Chromosomal transcriptional fusions. The DNA fragments containing the putative promoter regions of the *bapA, hvnA, pqsA, phnA, PA1215, PA1221, PA3662* and *glnK genes* were amplified by PCR, using appropriate oligonucleotide pairs (Table S6). The resulting DNA fragments were inserted into pCR2.1, excised by *Eco*RI/*Hin*dIII (*pqsA, phnA, PA1215, PA1221*), *Hin*dIII/*Bam*HI (*bapA, hvnA*), *Bam*HI/*Eco*RI (*glnK, PA3662*) digestions and inserted into the miniCTX-*lacZ* vector. These plasmids were used to generate chromosomal *bapA-lacZ, hvnA-lacZ, pqsA-lacZ, phnA-lacZ, PA1215-lacZ, PA1221-lacZ, PA3662-lacZ* *and glnK-lacZ* fusions, in the PAO1 or PAO1Δ*pprB* strains, as previously described (. The *FRT* cassette-excision step was not performed, resulting in the generation of strains maintaining tetracycline resistance.

Microarray design. The multi-genome *Pseudomonas aeruginosa* (MGPA) microarray used in this study has been designed based on the genome sequences of PAO1, PA14, PA7 and LESB58 strains available at [**http://www.pseudomonas.com**](http://www.pseudomonas.com/).For this MGPA microarray, eArray from Agilent was used to design a series of sixty base oligonucleotides (60-mer genomic markers) as follow: in a first step, all accessory and core genome genes of the four *P. aeruginosa* strains have been identified using systematic blast (BLAST 2) against genomes of the three other strains. In a second step, a unique specific sixty base oligonucleotide for each gene has been designed. In the case of paralogous genes, a **multiple alignment** was performed **by using ClustalW2 program to design** a unique and specific sixty base oligonucleotide for each paralog. This oligonucleotide design approach allowed us to distinguish subtle sequence differences among variants of orthologous and paralogous genes. At the end a total of 12333 oligonucleotides (including Agilent controls) representing unique sequences were randomly printed on the arrays in duplicate by using Agilent Sure-print technology in an 8x15K format.

Reverse transcription PCR (RT-PCR and RT-qPCR). Total cellular RNA was isolated, using the PureYield RNA Midiprep System (Promega). RT-PCR was carried out with the Access RT-PCR System (Promega), according the manufacturer's instructions, but modified by the addition of 6% DMSO to the RT-PCR mixture. For RT-PCR, a total of 1.8 µg of RNA or genomic DNA derived from the PprBK strain was used. RT-PCR was carried out with gene-specific primers overlapping junction regions between adjacent genes of the *bap* locus (listed in Table S6) with a T1 thermocycler (Biometra), using the following protocol: reverse transcription for 45 min at 45°C, inactivation of reverse transcriptase by incubation at 94°C for 2 min, followed by 45 cycles of PCR amplification with heating at 94°C for 30 s, 60°C for 1 min and 68°C for 2 min. We checked that RNA preparations were not contaminated with DNA, by carrying out the same experiment without adding the reverse transcriptase. The expected sizes of the amplicons for the various pairs of primers used were 1: 329 bp, 2: 398 bp, 3: 369 bp. For RT-qPCR, reverse transcription was performed on 2 µg of RNA by using the SuperScriptIII first strand synthesis system (Invitrogen). Cycling parameters of the real time PCR were 98°C for 2 min, following by 45 cycles of 98°C for 5 sec and 60°C for 10 sec, ending with 10 min at 95°C. To determine the amplification kinetics of each product (*pqsA, pelA, pslE, algD, cupE1, pprB,* and *amtB*), the fluorescence derived from the incorporation of EvaGreen into the double-stranded PCR products was measured at the end of each cycle using the SsoFast EvaGreen Supermix (Biorad). The Relative Expression Software Tool (REST) was used to calculate the relative expression of each gene under each condition using the *uvrD* gene as a reference for normalization, as well as the fold induction in the genes between an PprB-activated pathway condition versus its paired non activated reference condition .

Production and functionality of the PprB-His version. The His-tagged version of the PprB (PprB-6His) protein was produced in *E. coli* M15, after induction with 0.5 mM IPTG for 4 h at 28°C and was purified as recommended in previous studies . The PprB-6His version used for EMSA has been checked to be able to additionally complement a *pprB* mutant i/ for PprB-dependent expression of the reporter fusions *cupE–lacZ* (Fig. S6A, upper panel) and *flp–lacZ* (Fig. S6B, upper panel), as well as, ii/ for PprB-dependent production of CupE1 (Fig. S6A, lower panel) and Flp (Fig. S6B, lower panel) proteins. This demonstrates that PprB-6His used in the present study is a functional protein.

Production of antibodies directed against BapA. Two peptides from BapA, VGVDTDGDGQPDTTVV and DPSNGVELSGTAEPGV, were selected and synthesized and inoculated to rabbits at a concentration of 200 µg/ml. The immunization protocol included a first injection of the peptide, followed by three boosters, 15 days, one month and 2 months after the initial injection. Blood was subsequently collected from the rabbits; sera were isolated and purified further by incubation with the initial antigenic peptides. Pre-immune sera from the two rabbits were also checked for the absence of cross-reactivity with *P. aeruginosa* whole-cell extracts.

Bioinformatic analyses. The gene sequences are available from http://www.pseudomonas.com. The amino-acid sequences of the *hvnA and bapA* gene products were analyzed with softwares including Signal3P, SMART, ClustalW, PSI-pred, T-COFFEE all available at **http://www.embl heidelberg.de/predictprotein/predictprotein.html**. Additionally, BapA sequence was analyzed for tandem repeats in protein sequences using Xstream software .

**Microarray analysis.** Data were further treated with R (<http://cran.r-project.org/>) using LIMMA package from Bioconductor (<http://www.bioconductor.org/>). Intra-array normalization has been performed using local Lowess (Loess) regression method, whereas inter–array normalization has been performed using Aquantile method. Genes differentially expressed have been extracted using LIMMA package using linear regression (lmFit) and Bayesian models (eBayes) and considered as significant in the 4 replicates for a p-value<0.05. Lists of genes were further restricted applying a two-fold induction or repression criterium.

Resazurin tests. Quantitative evaluation of tobramycin toxic effect was performed using the resazurin test. 3 independent clones of each strain, PAO1 or PprBK were inoculated at initial 0.2 OD_600_ in 24-well plates for 24 hr at 30°C. 20 µg/ml of tobramycin were added for an additional 24 hr of incubation at 30°C. Non adherent cells were removed and biofilm forming bacteria were washed three times with water before being gently scrapped from the wells, resuspended in water to which resazurin was added at a final concentration of 20 µM for 24 hr. Fluorescence was measured using λ_exc_ and λ_em_ of 530 nm and 595 nm respectively.

References

1. Giraud C, Bernard CS, Calderon V, Yang L, Filloux A, et al. (2011) The PprA-PprB two-component system activates CupE, the first non-archetypal Pseudomonas aeruginosa chaperone-usher pathway system assembling fimbriae. Environ Microbiol 13: 666-683.

2. Bernard CS, Bordi C, Termine E, Filloux A, de Bentzmann S (2009) Organization and PprB-dependent control of the Pseudomonas aeruginosa tad Locus, involved in Flp pilus biology. J Bacteriol 191: 1961-1973.

3. Vasseur P, Vallet-Gely I, Soscia C, Genin S, Filloux A (2005) The pel genes of the Pseudomonas aeruginosa PAK strain are involved at early and late stages of biofilm formation. Microbiology 151: 985-997.

4. Byrd MS, Sadovskaya I, Vinogradov E, Lu H, Sprinkle AB, et al. (2009) Genetic and biochemical analyses of the Pseudomonas aeruginosa Psl exopolysaccharide reveal overlapping roles for polysaccharide synthesis enzymes in Psl and LPS production. Mol Microbiol 73: 622-638.

5. Kaniga K, Delor I, Cornelis GR (1991) A wide-host-range suicide vector for improving reverse genetics in gram-negative bacteria: inactivation of the blaA gene of Yersinia enterocolitica. Gene 109: 137-141.

6. Pfaffl MW (2001) A new mathematical model for relative quantification in real-time RT-PCR. Nucleic Acids Res 29: e45.

7. Wang Y, Ha U, Zeng L, Jin S (2003) Regulation of membrane permeability by a two-component regulatory system in Pseudomonas aeruginosa. Antimicrob Agents Chemother 47: 95-101.

8. Newman AM, Cooper JB (2007) XSTREAM: a practical algorithm for identification and architecture modeling of tandem repeats in protein sequences. BMC Bioinformatics 8: 382.

9. Smyth GK (2004) Linear models and empirical bayes methods for assessing differential expression in microarray experiments. Stat Appl Genet Mol Biol 3: Article3.
